# Morphological and molecular markers of mouse area CA2 along the proximodistal and dorsoventral hippocampal axes

**DOI:** 10.1002/hipo.23509

**Published:** 2023-02-10

**Authors:** Daniel Radzicki, Sarah Chong, Serena M. Dudek

**Affiliations:** ^1^ Neurobiology Laboratory, National Institute of Environmental Health Sciences National Institute of Health Research Triangle Park North Carolina USA

**Keywords:** cell types, hippocampal CA2, mossy fibers, PCP4, RGS14

## Abstract

Hippocampal area CA2 is a molecularly and functionally distinct region of the hippocampus that has classically been defined as the area with large pyramidal neurons lacking input from the dentate gyrus and the thorny excrescences (TEs) characteristic of CA3 neurons. A modern definition of CA2, however, makes use of the expression of several molecular markers that distinguish it from neighboring CA3 and CA1. Using immunohistochemistry, we sought to characterize the staining patterns of commonly used CA2 markers along the dorsal–ventral hippocampal axis and determine how these markers align along the proximodistal axis. We used a region of CA2 that stained for both Regulator of G‐protein Signaling 14 (RGS14) and Purkinje Cell Protein 4 (PCP4; “double‐labeled zone” [DLZ]) as a reference. Here, we report that certain commonly used CA2 molecular markers may be better suited for drawing distinct boundaries between CA2/3 and CA2/1. For example, RGS14+ and STEP+ neurons showed minimal to no extension into area CA1 while areas stained with VGluT2 and *Wisteria Floribunda* agglutinin were consistently smaller than the DLZ/CA2 borders by ~100 μ on the CA1 or CA3 sides respectively. In addition, these patterns are dependent on position along the dorsal–ventral hippocampal axis such that PCP4 labeling often extended beyond the distal border of the DLZ into CA1. Finally, we found that, consistent with previous findings, mossy fibers innervate a subset of RGS14 positive neurons (~65%–70%) and that mossy fiber bouton number and relative size in CA2 are less than that of boutons in CA3. Unexpectedly, we did find evidence of some complex spines on apical dendrites in CA2, though much fewer in number than in CA3. Our results indicate that certain molecular markers may be better suited than others when defining the proximal and distal borders of area CA2 and that the presence or absence of complex spines alone may not be suitable as a distinguishing feature differentiating CA3 from CA2 neurons.

## 1 INTRODUCTION

Recent work has highlighted the ways in which hippocampal area CA2 is both molecularly and functionally distinct when compared with the neighboring areas CA3 and CA1. Early studies, beginning with Lorente de Nó in 1934, distinguished the area that he named CA2 from area CA3 by the lack of “thorns” along the apical dendrites of *stratum lucidum* (SL), the layer superficial to the primary cell body layer (*stratum pyramidale* [SP]) which contains the presynaptic mossy fibers (MFs) of granule cells of the dentate gyrus (DG). This finding was confirmed by Ishizuka et al., and others, with Lorente de Nó's nomenclature updated to include the absence or presence of thorny excrescences (TEs) characteristic postsynaptic structures associated with MF synapses (Ishizuka et al., 1995). In rodents, CA2 neurons also exhibit distinct morphological, electrophysiological, and synaptic characteristics when compared with populations of primary excitatory neurons of area CA3 (Bartesaghi & Ravasi, 1999; San Antonio et al., 2014; Sun et al., 2017) and CA1 (Chevaleyre & Siegelbaum, 2010; Sun et al., 2021; Zhao et al., 2007). Beginning in 2014 with work published by Kohara et al. (2014), researchers began to appreciate that, in fact, a significant subset of the supposed CA2 neurons received functional excitatory synapses from the DG MFs in SL, reminiscent of the drawings of Ramón and Cajal (1899). Furthermore, while performing patch‐clamp recordings from CA2 pyramidal neurons, Kohara et al. (2014) found that the synaptic responses evoked with MF stimulation were significantly smaller in amplitude than those recorded from neurons located in area CA3. Retrograde and anterograde fluorescent labeling techniques have highlighted other major excitatory inputs to CA2, including inputs from CA3 (Cui et al., 2013), entorhinal cortex (Hitti & Siegelbaum, 2014; Lopez‐Rojas et al., 2022), the supramammillary (SuM) nucleus of the hypothalamus (Chen et al., 2020; Cui et al., 2013), and cholinergic input from the medial septum (Cui et al., 2013). Interestingly, Hitti & Siegelbaum (2014) found no evidence of MF input to CA2 using rabies‐based labeling techniques hinting that this method may be limited when labeling DG input to area CA2.

Several proteins and genes such as neurotrophin‐3 (NT3), basic fibroblast growth factor (bFGF/FGF2), and alpha‐actinin 2 had been noted to be enriched in CA2 (Eckenstein et al., 1991; Phillips et al., 1990; Wyszynski et al., 1998; Young et al., 2006). However, as interest in CA2 has grown, antibodies against proteins such as the Striatal Enriched Protein Tyrosine Phosphatase (STEP; Boulanger et al., 1995), N‐terminal EF‐hand Calcium‐Binding protein 2 (Necab2) (Zimmermann et al., 2013), Purkinje Protein 4 (PCP4; San Antonio et al., 2014), and Regulator of G‐protein Signaling 14 (RGS14; Lee, Simons, 2010) have made the identification of area CA2 less controversial. In addition, other markers of CA2 pyramidal neurons include the plant lectin *Wisteria Floribunda* agglutinin (WFA) to label the specialized extracellular matrix called perineuronal nets (PNNs; Bruckner et al., 2003; Carstens et al., 2016) and antibodies for the vesicular glutamate transporter 2 (VgluT2) to label the hypothalamic inputs to CA2 neurons originating from SuM projection neurons (Chen et al., 2020; Herzog et al., 2006) have been used to distinguish CA2 from neighboring subfields. Last, spatially restricted RNAseq‐based approaches have been used to compare and contrast the unique molecular heterogeneity of hippocampal neurons along the proximodistal CA axis and allow researchers to further compartmentalize transcriptomal differences based on somatic and dendritic subregions (Cembrowski, Bachman, et al., 2016; Cembrowski, Wang, et al., 2016; Farris et al., 2019). Gene expression patterns can also be used to better define the anatomical boundaries of area CA2 in combination with axonal input/output patterns along the longitudinal hippocampal axis as detailed in this issue's commentary by Bienkowski (2023). Though not typically used as markers, expression of the vasopressin 1b receptor (Avpr1b), which is selectively expressed in CA2, and the oxytocin receptor, expressed in CA2 and distal CA3, sparked research into CA2's role in social behavior (Hitti & Siegelbaum, 2014; Raam et al., 2017; Tirko et al., 2018), for example.

The distinct gene expression in CA2 has made possible many studies on CA2's function as well. For example, cellular activity and synaptic transmission can be modulated in vivo via the expression of Green Fluorescent Protein (GFP) or effector proteins using Cre recombinase under the control of CA2‐specific promoters like *Amigo2* (Alexander et al., 2018; Hitti & Siegelbaum, 2014; Smith et al., 2016), *Cacng5* (Boehringer et al., 2017; Shinohara 2012), and *Map3k15* (Kohara et al., 2014), which have implicated the role of CA2 neuronal activity in fundamental behaviors like social processing (Alexander et al., 2016; Hitti & Siegelbaum, 2014; Meira et al., 2018; Smith et al., 2016), aggression (Leroy et al., 2018; Pagani et al., 2015), hippocampal‐dependent memory (Alexander et al., 2019; Boehringer et al., 2017; Chen et al., 2020) and spatial coding (Alexander et al., 2016; Kay et al., 2016).

Given the myriad of molecular tools available, we set out to further investigate the morphological features associated with the MF CA2 synapses and the distinct molecular characteristics of hippocampal area CA2, in relation to the neighboring CA subfields, to better understand both how best to define cellular heterogeneity in this region. In addition, we sought to gain a clearer understanding of the degree to which commonly used CA2 immunohistochemical markers colocalize in the proximal and distal borders of hippocampal area CA2.

## 2 MATERIALS AND METHODS

### 2.1 Animals and handling

All procedures were approved by the NIEHS Animal Care and Use Committee and were in accordance with the National Institutes of Health guidelines for care and use of animals. Wild‐type adult mice, either c57BL/6J (Jax 000664) or bred on a c57BL/6J background, were used for all experiments. Male and female adult mice (13–22 weeks old) were used for immunofluorescence experiments. Both male and female adult mice were used for viral injection experiments.

### 2.2 Intracranial injections of viral vectors

Adult mice were temporarily anesthetized using isoflurane (0.5% at 0.8 L/min) and then received and intraperitoneal injection of a ketamine/xylazine cocktail (10 and 0.7 mg/mL, respectively) at 0.1 cc/10 g body weight. Ophthalmic ointment was then applied to the eyes and the scalp was shaved and treated with two applications of betadine. Lidocaine (0.05 cc of 0.5% Marcaine) was injected subcutaneously. After being secured in the stereotaxic instrument, an incision was made along the midline exposing the skull surface and after removing the dura, pilot holes were unilaterally drilled above area CA2 (‐2.4 ML, ‐2.3 AP, and ‐1.9 DV) and DG (‐1.15 ML, ‐2.0 AP, ‐1.9 DV, all coordinates relative to bregma). Virus was infused into either CA2 (300 nl of AAV5.hSyn.GCamp6f; Addgene 100837; at a rate of 100 nl/min) or into the DG (250 nl of AAV5.hSyn.ChR2.mcherry; Addgene 26976; 50 nl/min) using a Hamilton syringe position in a syringe pump (KD Scientific) and tube‐fed cannulas made in house from blunted 26G needles (BD). The cannula was left in position for a minimum of 8 min before a slow retraction. The scalp was then sutured, and Buprenorphine‐SR was administered subcutaneously (SQ; 0.005 mg/mL, 0.02 cc/10 g body weight) followed by the antisedant Atipamezole (0.2 mg/kg, 0.1 cc dosed SQ). Animals were allowed to recover on a warming pad until regaining consciousness and returning to the home cage. Animals remained in their home cages for a minimum of 2.5 weeks to allow for the proteins to express prior to tissue collection.

### 2.3 Tissue processing and immunohistochemistry

Mice were fully anesthetized with Fatal Plus (sodium pentobarbital, 50 mg/mL: >100 mg/kg) via intraperitoneal injection. Immediately after transcardial perfusion with 4% paraformaldehyde (pH 7.4) in phosphate buffered saline (PBS), brains were removed and post‐fixed in the same fixative overnight at 4°C. Brains were sectioned at 40 μm, at an angled horizontal plane (Bischofberger et al., 2006) on a Leica VT 1200S vibratome. Hippocampal sections were stored free floating in PBS with 0.02% sodium azide at 4°C.

All free‐floating sections were incubated in 5% normal goat serum in PBS for 1 h, prior to incubation in primary antibodies overnight, rocking at 4°C. After washing 3× 10 min in PBS with 0.01% Triton X‐100, sections were incubated in secondary antibodies for 2 h at room temperature. Sections were washed again for 3× 15 min and cover slipped with Vectashield Hardset Mounting Medium with DAPI (Vector, H‐1500‐10).

The following antibodies were used to label RGS14 and PCP4 in most experiments: mouse anti‐RGS14 (1:500, NeuroMab, 75‐170), rabbit anti‐PCP4 (1:500, Invitrogen, PA5‐52209), Alexa Fluor 568 secondary (goat anti‐mouse, 1:500, Invitrogen, A11004), and Alexa Fluor 488 secondary (goat anti‐rabbit, 1:500, Invitrogen, A11034). Antibodies not mentioned here are summarized in Table 1 and listed by experiment.

**TABLE 1:** Primary and secondary antibodies used in immunohistochemical experiments for indentifying molecular markers of area CA2

Experiment	Primary antibody	Secondary antibody
STEP × RGS14 × PCP4	Anti‐STEP (mouse isotype IgG1, 1:500, Millipore 05‐730, RRID:AB_2173551); anti‐RGS14 (mouse isotype2a, 1:500, NeuroMAB, 75‐170. RRID:AB_2179931); anti‐PCP4 (1:500, Invitrogen PA5‐52209, RRID:AB_2645298)	Alexa488 goat anti‐mouse IgG1 (1:500, Invitrogen A21121, RRID:AB_2535764); Alexa555 goat anti‐mouse IgG2a (1:500, Invitrogen A21137, RRID:AB_2535776); CF633 goat anti‐rabbit (1:500, Biotium 210123, RRID:AB_10563033)
VGluT2 × RGS14 × PCP4	Anti‐VGluT2 (guinea pig, 1:10,000, Millipore AB225‐1, RRID:AB_2665454); anti‐RGS14 (mouse isotype2a, 1:500, NeuroMAB, 75‐170. RRID:AB_2179931); anti‐PCP4 (1:500, Invitrogen PA5‐52209, RRID:AB_2645298)	Alexa488 goat anti‐guinea pig (1:500, Invitrogen A11073, RRID:AB_2534117); Alexa555 goat anti‐mouse IgG2a (1:500, Invitrogen A21137, RRID:AB_2535776); CF633 goat anti‐rabbit (1:500, Biotium 210123, RRID:AB_10563033)
WFA × RGS14 × PCP4	Wisteria Floribunda Lectin, Biotinylated (1:1,000, Bl‐355, Vector); anti‐RGS14 (mouse isotype2a, 1:500, NeuroMAB, 75‐170. RRID:AB_2179931); anti‐PCP4 (1:500, Invitrogen PA5‐52209, RRID:AB_2645298)	Alexa633 streptavidin (521375, 1:500, Invitrogen); Alexa555 goat anti‐mouse IgG2a (1:500, Invitrogen A21137, RRID:AB_2535776); CF633 goat anti‐rabbit (1:500, Biotium 210123, RRID:AB_10563033)
Aggrecan × RGS14	Anti‐Aggrecan (rabbit, 1:500, AB1031, Millipore); anti‐RGS14 (mouse isotype2a, 1:500, NeuroMAB, 75‐170. RRID:AB_2179931)	Alexa568 goat anti‐rabbit (A11004, 1:500, Invitrogen); Alexa488 goat anti‐mouse (Invitrogen A11059, RRID:AB_142495)
Znt3 × RGS14	Anti‐Znt3 (guinea pig, 1:500, 197004, Synaptic Systems); anti‐RGS14 (mouse isotype2a, 1:500, NeuroMAB, 75‐170. RRID:AB_2179931)	Alexa568 goat anti‐guinea pig (A11075, 1:500, Invitrogen); Alexa488 (goat anti‐mouse, Invitrogen A11059, RRID:AB_142495)

Abbreviation: IgG, Immunoglobulin G.

### 2.4 Microscopy and analysis

All images were acquired with an LSM 880 Zeiss confocal microscope. All quantifications were made on maximum intensity Z‐projected images with ImageJ (Schneider et al., 2012). Statistical significance was calculated using an unpaired, two‐tailed *t* test assuming equal variance and a Gaussian distribution. If distributions proved to be unequal, a Welch's *t* test was calculated and reported as such in the text. Data are presented as means ± SEM.

For analysis of the RGS14+/PCP4+ double‐labeled zone (DLZ) and other commonly used markers of CA2, 3D tile scans (20× objective, 1024 × 1024 pixels) were obtained at 0.35 μm (Z‐depth) increments to include all labeled RGS14 and PCP4 neurons of the *stratum pyramidale* (SP) in CA1‐3. Dorsoventral depths corresponding approximately to the following figures from Paxinos and Franklin's mouse brain atlas (Paxinos & Franklin, 2012) were used for dorsoventral analyses: Figures 156 (dorsal), 153 (intermediate), and 147 (ventral). Only hippocampal sections displaying consistent fluorescence intensities for all immunohistochemical stains were imaged, and consistent image capture settings were used to image all sections. For analysis of just the RGS14+/PCP4+ DLZ, two or three sections per dorsoventral depth for 17, 15, and 13 (dorsal, intermediate, ventral, respectively) animals were imaged. For quantification of WFA, two or three sections per dorsoventral depth of three animals were imaged. For quantification of VGluT2, three sections per dorsoventral depth from five animals were imaged. For analysis of Aggrecan (ACAN) and STEP, four sections per dorsal depth from two animals were imaged.

RGS14+/PCP4− and PCP4+/RGS14− cells were observed along the proximodistal hippocampal axis. Continuous immunohistochemical staining was defined as positively labeled cells being no more than 200 μm apart. Accordingly, imaging windows included at least 200‐μm beyond each border of continuous labeling. Measurements of each individual stain (e.g., just RGS14) were made using a magnification level of 150%, by an investigator blinded to just one stain. All counts for continuously labeled cell bodies were restricted to the SP and measured along the midline of SP in CA1‐3. We defined the CA1‐subiculum border as the distal‐most end of the compact pyramidal cell layer. Because the SP‐*stratum oriens* (SO) border of distal CA1 is too diffuse to delineate objectively, the width of the SP was restricted throughout the more distal CA1 to match the width of the widest portion of the CA1 compact pyramidal cell layer.

To determine the RGS14+/PCP4+ DLZ, the overlap of RGS14 and PCP4 was then visually inspected for co‐expression. Within the length of RGS14/PCP4 overlap, the borders of the DLZ were defined by the distal‐most and proximal‐most cells that show double‐labeling of RGS14 and PCP4.

For quantification of mossy fiber boutons (MFBs), 3D Z‐series stacks (40× oil immersion objective, 2048 × 2048 pixels) were obtained at 0.64‐μm increments along the SL of the CA2‐CA3 axis. Two dorsal and two intermediate sections (corresponding to Figures 156 and 153, respectively) each from all three animals were imaged 8.5‐μm thick maximum intensity projections centered on the SL of CA2 were generated and quantified at a magnification level of 75% in ImageJ. All mCherry‐positive MFBs within the SL were drawn manually, along the midline of the SL, as a function of distance from the distal end of *stratum lucidum*. ImageJ was used to measure the area (μm^2^) of each hand‐drawn MFB. Large MFBs were characterized by a cross‐sectional area of at least 4 μm^2^, equivalent to the minimum volume (6 μm^3^) characteristic of giant MFBs (LaSarge et al., 2015). Small boutons were characterized by a maximum cross‐sectional area of 2 μm^2^, and medium boutons were characterized by a cross‐sectional area of 2.01 to ~3.99 μm^2^. For comparisons of MFB density in dorsal versus intermediate CA2, MFB density was normalized to the area (μm^2^) within the distal‐most 50 and 100 μm of the SL.

## 3 RESULTS

### 3.1 MFB innervation along the hippocampal proximodistal and rostrocaudal axes

Early investigations into hippocampal neuronal morphology by Lorente de Nó defined area CA2 as distinct region of Cornu Ammonis (CA) characterized by pyramidal neurons that lacked innervation from MF axons originating from granule cells of the DG, as well as an absence of complex spines, or thorns/TEs, seen on apical dendrites in CA3 neurons (Dudek et al., 2016; Ishizuka et al., 1995; Lorente de Nó, 1934). More recent work has refined this definition by demonstrating that a large subset of CA2 pyramidal neurons, as defined by staining for PCP4 (San Antonio et al., 2014), receive synaptic input from MF axons, though lack complex spines (Kohara et al., 2014). Using intracranially injected, virally expressed fluorescent constructs to label both presynaptic (MFs) and postsynaptic principal neurons, we sought to further characterize the morphology and distribution of MFBs at high resolution (40× and 80× magnification) along the proximodistal CA3/CA2 axis in the mouse hippocampus. Moreover, given the rostrocaudal spread of the CA region, we further subdivided our analysis along the dorsal–ventral axis in horizontal sections (Bischofberger et al., 2006; Supplementary S1).

**FIGURE 1:**
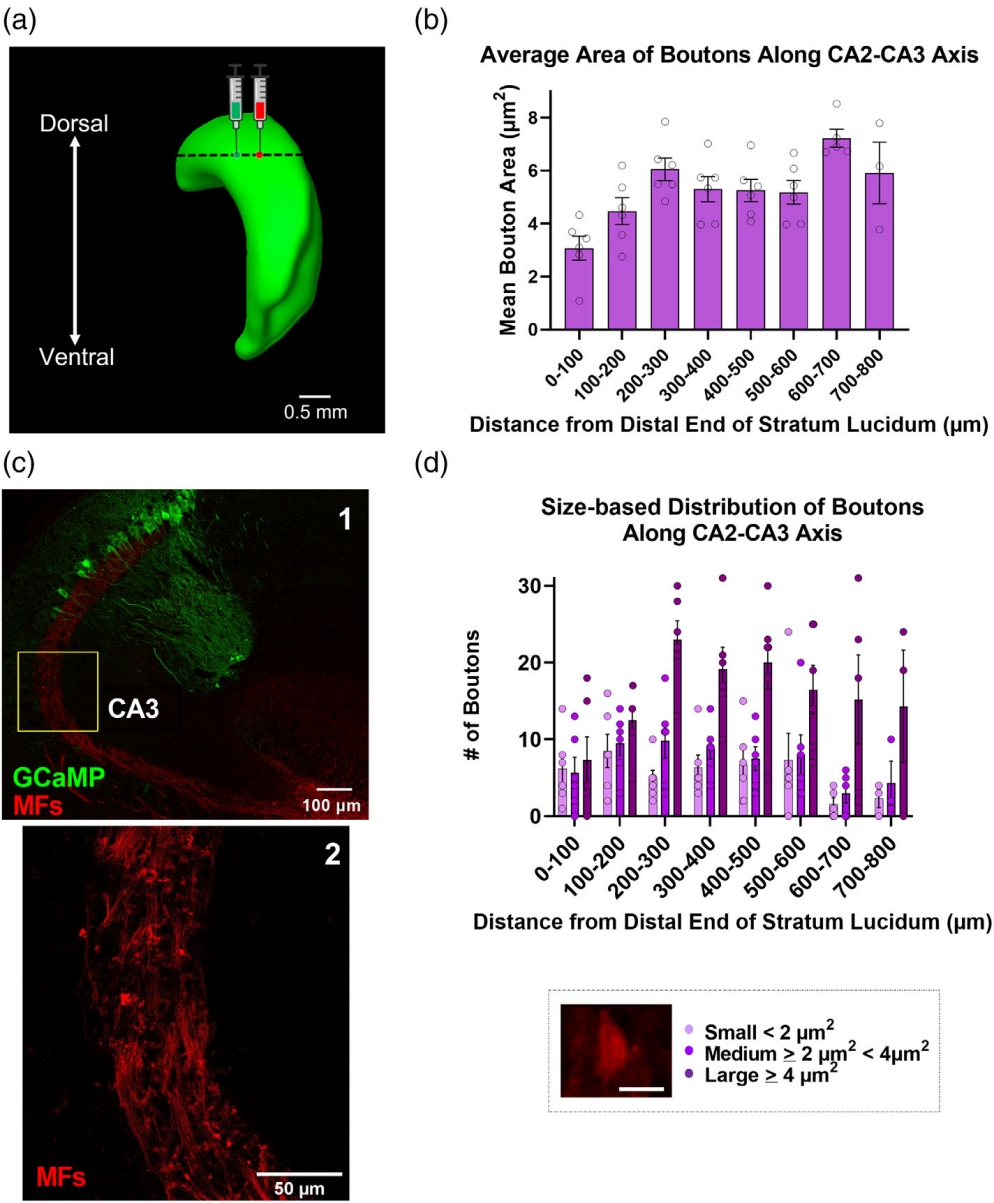
Mean area of mossy fibers boutons originating from the dorsal DG increases along the CA2 → CA3 axis. (a) Viral injection scheme depicting intracranial injections of AAV5.hSyn.ChR2.mcherry and AAV5.hSyn.GCaMP6f into dentate gyrus and CA2, respectively, in dorsal hippocampus (lateral view) in wild‐type c57/B6J mice. (b) Mean area of mossy fiber boutons increases along the CA2 → CA3 axis, moving away from CA1 (3.63 ± 2.33 μm^2^ vs. 5.07 ± 0.27 μm^2^ within 0–100 and 500–600 μm, respectively, of the distal end of *stratum lucidum* towards CA3, *p* = .008, *t* = 3.32, *f* = 1.02, *n* = 6 sections from 3 mice). (c1) Reference overview image (40× magnification) of a horizontal section from dorsal hippocampus, at dorsal injection site, showing GCaMP‐labeled CA2/CA3 pyramidal neurons and mCherry‐labeled mossy fibers. (c2) Expanded view (40− magnification; 2048 × 2048 pixels) of inset in (c1), showing hand‐drawn ROIs for well‐isolated boutons in CA3, 450–650 μm from dSL. (d) Ratio of large boutons to small boutons increases from CA2 to CA3, disto‐proximally, moving away from CA1 (0.159 vs. 0.895 within 0–100 and 500–600 μm, respectively, of the dSL, *n* = 6 sections from 3 mice). Scale bar = 3.5 μm. Values are presented as mean ± SEM.

MFs originating from rostrodorsal granule cells of the DG were labeled via intracranial injections of AAV encoding red fluorescent protein (AAV5‐ChR2‐mcherry), while a second injection containing (non‐Cre dependent) GCamp6f to label both CA2 and distal CA3 (CA3d) pyramidal neurons was targeted to rostrodorsal area CA2 (Figure 1a,c). MFBs were identified manually in 40× confocal images with individual bouton areas and bouton distances plotted relative to the distal‐most end of s*tratum lucidum* (*dSL*), towards CA1, and analyzed using ImageJ software (Figure 1, c2). We found a nonsignificant reduction in the number of labeled MFBs along the proximodistal axis of CA3 → CA2 (in the direction of CA1, terminating at the end of *stratum lucidum* [*dSL*]), with a 166% reduction in boutons when comparing the CA3 region 500–600 μm from dSL, towards CA3, with the 100 μm adjoining the dSL (i.e., mid CA2; Figure S2). Consistent with this finding, the average bouton area significantly decreased along the CA3 → CA2 axis, as one moves towards the distal tip of the SL. Distal boutons within 100 μm of dSL (CA2) were on average significantly smaller than boutons located 500–600 μm from dSL (towards CA3; Figure 1b, 3.634 ± 2.325 and 5.073 ± 0.274 μm^2^, *p* = .0077, *n* = 6, 6 sections, respectively from 3 animals). Last, identified boutons were divided into small (<2 μm^2^), medium (≥2 μm^2^ <4 μm^2^), and large (≥4 μm^2^) groupings. All three categories were equally represented in the distal‐most 100 μm of SL (CA2, towards CA1) with a large/small ratio of 1.2/1 (Figure 1d, 0–100 μm bin, dark vs. light purple bars). In contrast, large boutons were most abundant in the more proximal, CA3‐associated SL, with a large/small ratio of 9.5/1 in the region 600–700 μm from dSL towards CA3 (Figure 1d, 600–700 μm bin, dark vs. light purple bars).

**FIGURE 2:**
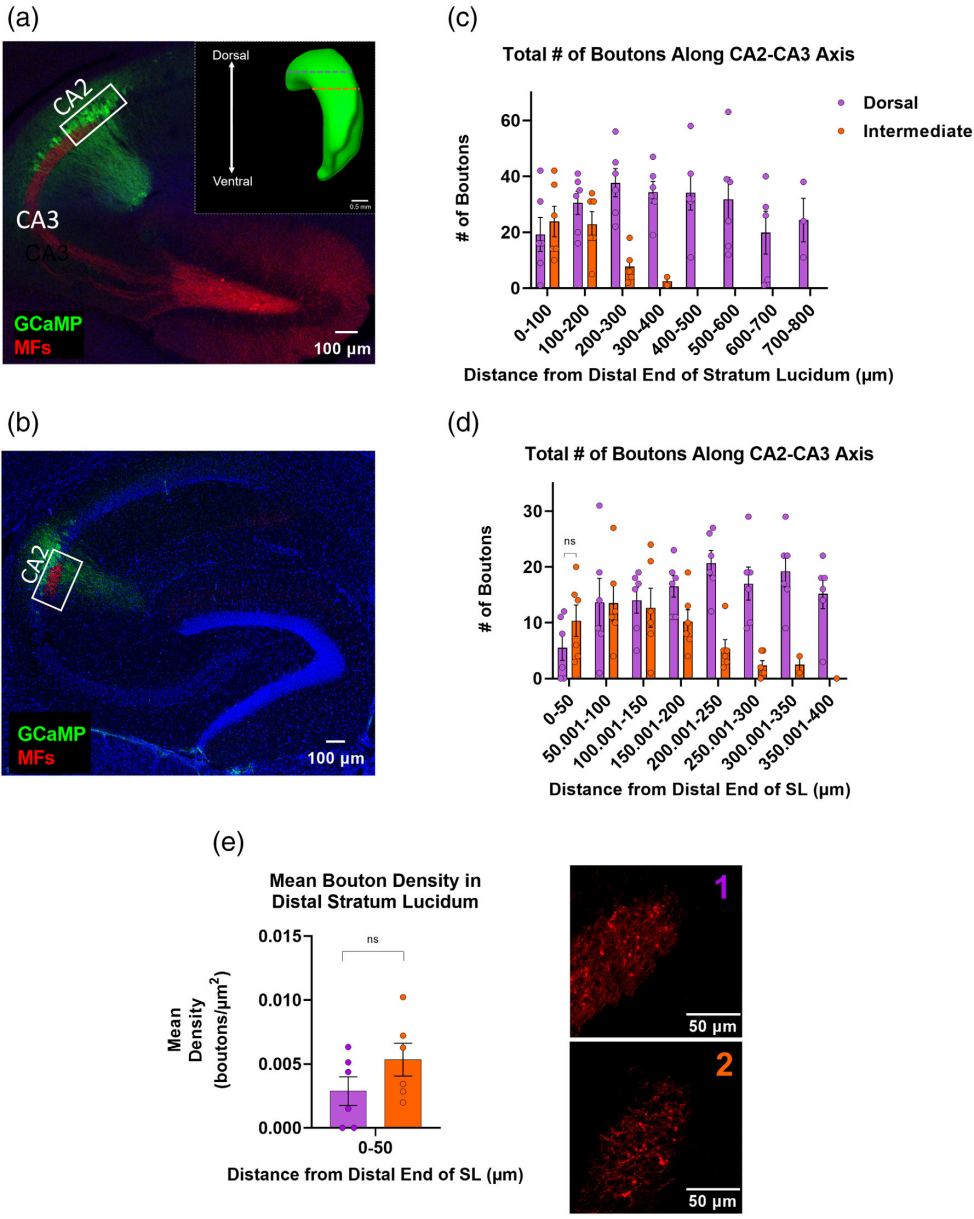
Mossy fiber (MF) boutons originating from rostrodorsal dentate gyrus (DG) appear to preferentially target intermediate CA2 over the neighboring, dorsal CA3 region. (a) Horizontal section from dorsal hippocampus, at dorsal injection site, showing GCaMP‐labeled CA2/CA3 pyramidal neurons (green) and mCherry‐labeled DGGCs (red). Inset: Lateral view of hippocampus depicting dorsal and intermediate depths for analysis of mossy fiber bouton (MFB) morphometrics. (b) Horizontal section from intermediate hippocampus exhibiting CA2‐specific innervation by mossy fibers of the dorsal DG. (c) Total number of boutons as a function of distance from the distal end of *stratum lucidum*, towards CA3, in dorsal and intermediate sections (*n* = 6 dorsal sections and *n* = 6 intermediate sections from three mice). (d) Total number of MFBs is statistically similar in dorsal versus intermediate CA2 at the distal‐most 50 μm of the stratum lucidum (dorsal CA2: 5.5 ± 2.25 MFBs, intermediate CA2: 10.33 ± 2.84 MFBs, *p* = .21, *t* = 1.34, *f* = 1.59, *n* = 6, 6 sections, respectively from 3 animals). (e) Mean bouton density is similar in the distal most 50 μm of *stratum lucidum* in dorsal sections (a and e1) versus intermediate (b and e2) CA2 (0.00289 ± 0.00112 and 0.00534 ± 0.00128 boutons/μm^2^, *p* = .18, *t* = 1.44, *f* = 1.31, *n* = 6, 6 sections, respectively, from 3 animals).

Previous work has described an interesting phenomenon wherein MF axons originating from dorsal granule cells project in the horizontal plane to contact nearby CA3 and CA2 neurons but then continue ventrolaterally within CA2 and seem to target area CA2 exclusively (Anderson, 2007; Kohara et al., 2014). This projection can also be visualized in single granule cells filled and imaged by the Janelia Mouselight Project (ml-neuronbrowser.janelia.org). To further characterize the individual boutons of these distinct projections, we again labeled both presynaptic boutons (red) and postsynaptic neurons (green) and identified individual boutons in the horizontal plane (Figure 2a,b). As expected, boutons were most densely associated within the distal end of *stratum lucidum* (CA2; 0–100 and 100–200 μm bins) but then fell off dramatically in the more proximal regions of CA3 in the more ‘intermediate’ sections ventral to the viral injection site (Figure 2c). Next, we compared boutons in the distal‐most 50 μ of *stratum lucidum* (within area CA2) in both dorsal sections (Figure 2a) and the more intermediate hippocampal sections, ventral to the labeled MF cell bodies, that exhibit this CA2‐specific axonal targeting (Figure 2b) but found no statistical difference in either mean total number of boutons (Figure 2d; 5.5 ± 2.247 and 10.33 ± 2.84 MFBs, *p* = .2113, *n* = 6, 6 sections, respectively from 3 animals) or mean bouton density (Figure 2e, 0.003 ± 0.0011 and 0.0053 ± 0.0013 boutons/μm^2^, *p* = .181, *n* = 6, 6 sections, respectively). We note that no boutons were observed in CA3 at distances >400 μm from dSL in intermediate sections ventral to the viral injection site.

**FIGURE 3:**
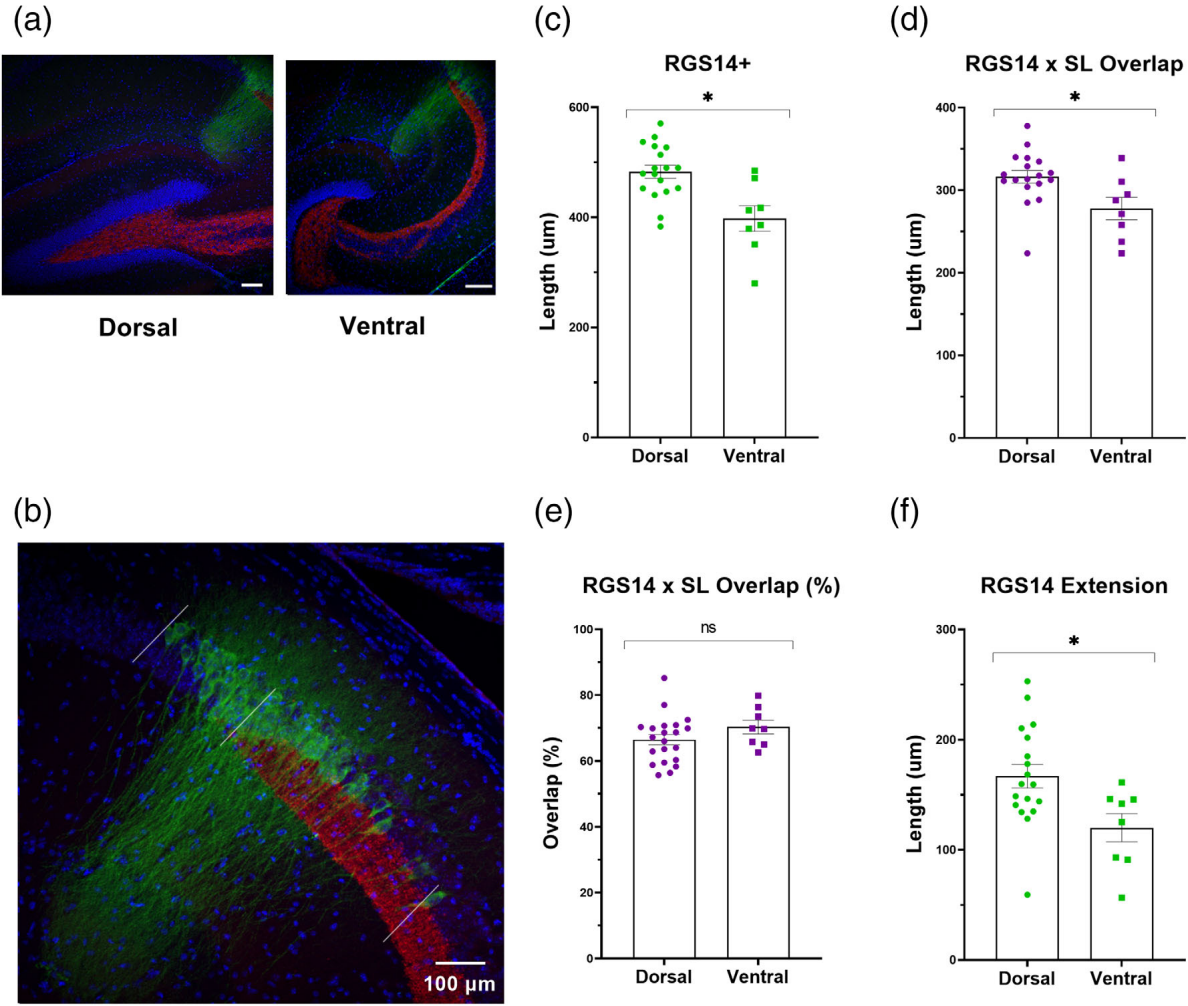
RGS14 expression patterns in relation to Znt3+ mossy fibers (MFs) differ along the dorsal–ventral axis of the hippocampus. (a) Slice position along the dorsal–ventral axis was confirmed by the shape of the dentate gyrus in 10× confocal images as illustrated in the left (dorsal, “V‐shape”) and right (ventral, “C‐shape”) panels. Scale bars = 200 μm (b) 20× confocal image comprising the extent of RGS14 labeling relative to Znt3+ staining. The length between the proximal and distal extent of RGS14+ immunohistochemical staining (outside white lines) was subdivided based on proximity to the distal end of the Znt3+ *stratum lucidum* (center white line). Scale bar = 100 μm (c) The length of RGS14+ expression is significantly larger in dorsal versus ventral slices (483.14 ± 11.70 vs. 397.95 ± 23.18 μm, *p* = .001, *t* = 3.66, *f* = 1.74, *n* = 18, 8 sections). (d) On average, the length of RGS14+ staining (green, b) that overlaps with labeled mossy fibers in stratum lucidum (red, b) is significantly longer in dorsal versus ventral slices (316.24 ± 7.58 μm in dorsal slices and 277.83 ± 13.49 in ventral, *p* = .014, *t* = 2.66, *f* = 1.41, *n* = 18, 8 sections, from 8 animals). (e) The percent overlap, relative to total RGS14+ length, is, on average, similar in dorsal and ventral sections (66.45% and 70.34%, *p* = .19, *t* = 1.34, 1.48, *n* = 18, 8 sections, from 8 animals). (f) The length of RGS14+ staining that extends beyond the distal most tip of *stratum lucidum* (towards CA1) is significantly longer in dorsal hippocampal slices when compared with ventral slices (166.91 ± 10.77 and 120.13 ± 12.77 μm, *p* = .017, *t* = 2.55, *f* = 1.6, *n* = 18, 8 sections, from 8 animals).

In an effort to further characterize the relationship between MF axons and area CA2, we used immunohistochemistry (IHC) to label Zinc transporter 3 (Znt3) positive MFs and RGS14 positive neurons in both dorsal and ventral horizontal hippocampal sections (Figure 3a,b). When measuring the extent of RGS14 positivity, we observed significantly more labeling in dorsal sections when compared with more ventral sections (Figure 3c, 483.14 ± 11.70 vs. 397.95 ± 23.18 μm, *p* = .001, *n* = 18, 8 sections from 8 animals). On average, the RGS14+ overlap with Znt3+ *stratum lucidum* measured 316.24 ± 7.58 μm in dorsal sections, which was significantly greater than in ventral hippocampus (dorsal 316.24 ± 7.58 vs. ventral 277.83 ± 13.49 μm, *p* = .014; Figure 3d: *n* = 18, 8 sections from 8 animals). Interestingly, the percent overlap of the two markers across the D‐V axis was similar at both levels implying that reduction in total RGS14 length is, on average, proportional to the reduction in RGS14+/Znt3+ overlap in dorsal and ventral sections (Figure 3e, 66.50% for dorsal and 70.34% for ventral, *p* = .19, *n* = 18, eight sections from eight animals). The remaining ~30% of RGS14+ neurons did not overlap with Znt3+, marking a subset of CA2 neurons that are unlikely to receive direct synaptic input from granule cells, and this fraction was also significantly greater than in ventral sections (Figure 3f, ventral 166.91 ± 10.77 and 120.13 ± 12.77 μm, *p* = .017, *n* = 18, 8 sections from 8 animals).

**FIGURE 4:**
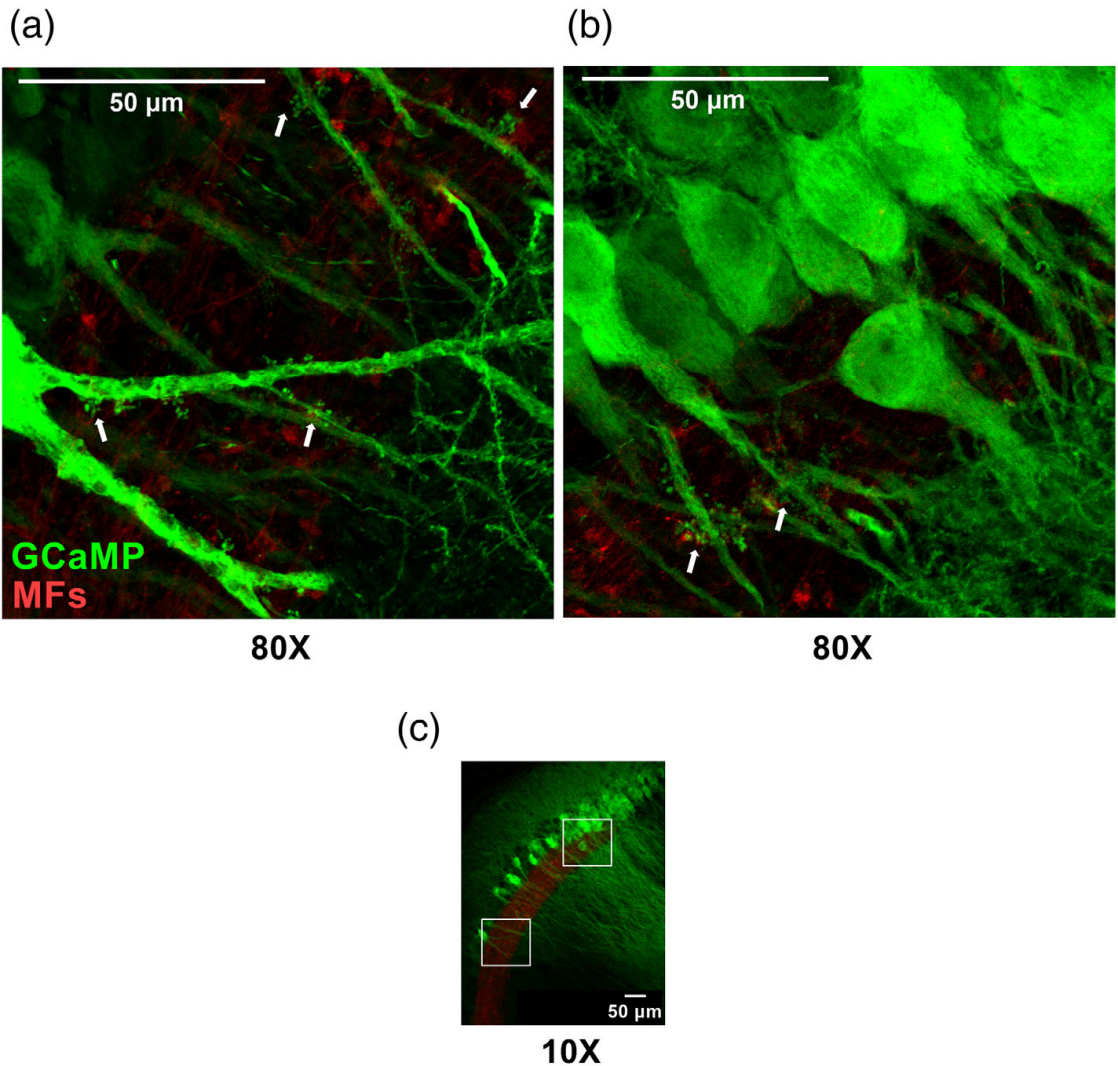
Some apical dendrites of CA2 pyramidal neurons display thorny excrescences (TEs) in *Stratum Lucidum*. (a,b) Z‐projected confocal images (80×) showing TEs of the apical dendrites of CA3 pyramidal neurons (A), located >400 μm from dSL, and CA2 pyramidal neurons (b), located within 180 μm of dSL. White arrows indicate TEs. (c) Reference overview image (10× magnification) with insets depicting location of expanded views from (CA3, a) and (CA2, b).

Last, one benefit of labeling postsynaptic neurons in a noncell‐type‐specific manner (independent of Cre‐recombinase) was that a single injection labeled both “off‐target” CA3 and targeted CA2 neurons, allowing us to visualize postsynaptic morphologies along the proximodistal CA3 → CA2 axis (towards CA1), including cells that both were and were not aligned with *stratum lucidum*. Our interpretation of the imaged location was further aided by the RGS14+ measurements reported in Figure 3. As expected, the apical dendrites of putative CA3 neurons, located >400 μm from dSL (towards CA3), contained complex postsynaptic clusters, TEs (Figure 4a, white arrows). As might have also been expected, TEs were absent in GCaMP‐labeled neurons located just beyond dSL (CA2 neurons closer to CA1; Supplementary S3), a region that consistently shows strong RGS14 labeling (Figure 3b,f) but lacks MF input. Surprisingly though, we did observe examples of these complex spine structures in CA2 neurons located within 200 μm of the distal‐most tip of *stratum lucidum* towards CA3, though such excrescences uncommon (Figure 4b, white arrows and Supplementary S3). Although these CA2‐associated complex spines are sparse, they do hint at heterogeneity within the CA2 neuronal population regarding the presence of complex MF spines.

### 3.2 RGS‐14 and PCP4 immunopositivity in areas CA3, CA2, and CA1

**FIGURE 5:**
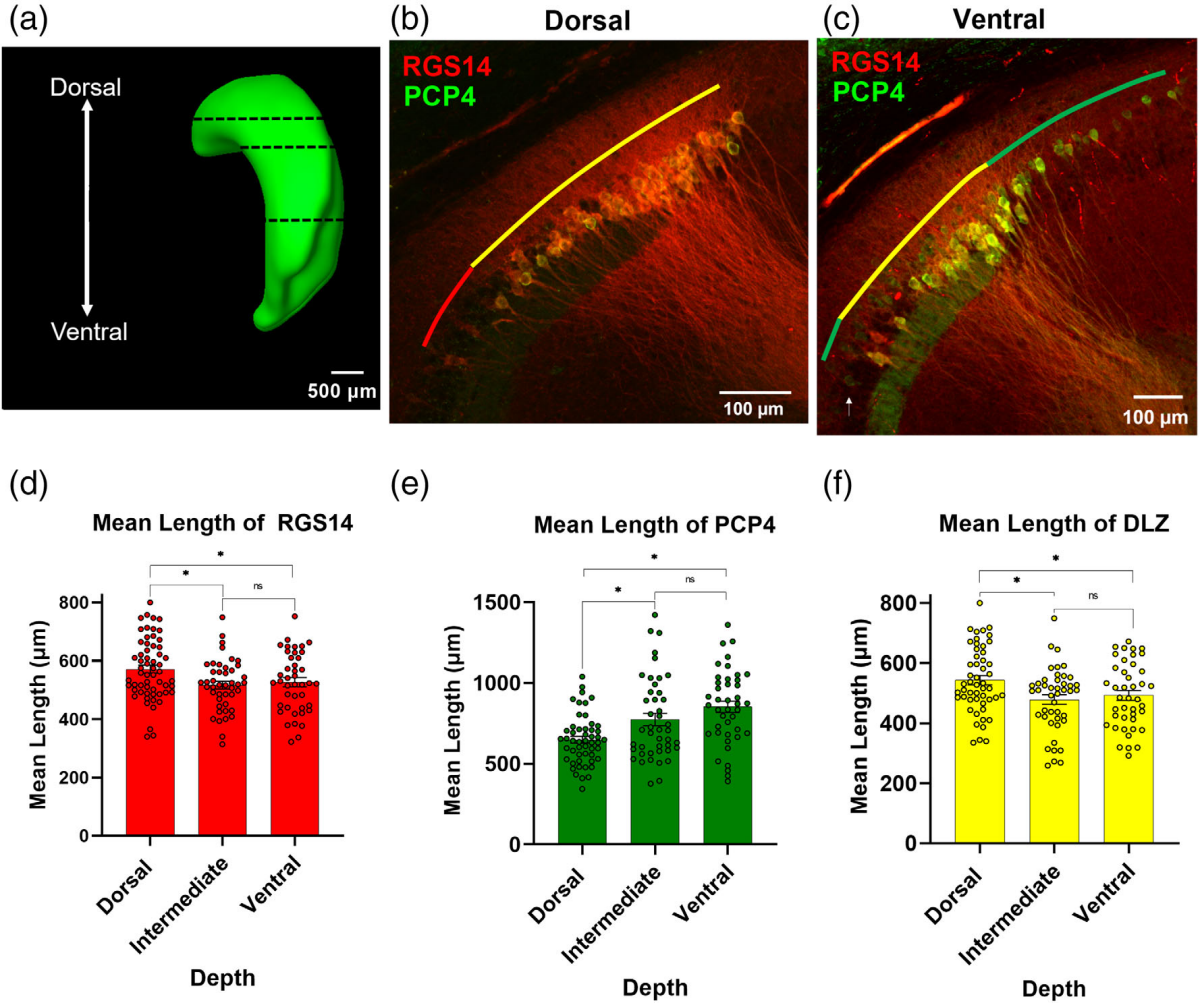
RGS14 and PCP4 staining overlaps along the dorsoventral axis, with the greatest overlap being in dorsal hippocampus. (a) Lateral view of hippocampus depicting dorsal, intermediate, and ventral depths for analysis of all immunohistochemical markers. (b,c) Representative images of RGS14 and PCP4 immunofluorescence. Colored lines indicate the RGS14^+^/PCP4^+^ double‐labeled zone (DLZ) in yellow, with RGS14 (red) and PCP4 (green) extension beyond the DLZ, in dorsal (b) and ventral (c) depths. (d) Mean length of RGS14 labeling is largest in dorsal levels (dorsal: 571.25 ± 13.58 μm; intermediate: 516.56 ± 13.29 μm; ventral: 526.04 ± 15.75 μm, *n* = 59, 44, 42 sections, from 17, 15, 13 animals, respectively; dorsal vs. intermediate *p* = .006, *t* = 2.81, *f* = 1.4; intermediate vs. ventral *p* = .65, *t* = 0.46, f=1.34, dorsal vs. ventral *p* = .033, *t* = 2.17, *f* = 1.04). (e) Mean length of PCP4 labeling is greatest in ventral depths (dorsal: 648.98 ± 21.42 μm; intermediate: 774.23 ± 38.44 μm; ventral: 852.96 ± 35.46 μm *n* = 51, 44, 42 sections, from 17, 15, 13 animals, respectively; dorsal vs. intermediate Welch's *p* = .006, Welch's corrected *t* = 2.85, *f* = 2.78; intermediate vs. ventral *p* = .14, *t* = 1.50, *f* = 1.23, dorsal vs. ventral Welch's *p* ≤ .0001, Welch's corrected *t* = 4.92, *f* = 2.26). (f) Mean length of DLZ is largest in dorsal levels (dorsal: 543.20 ± 15.34 μm; intermediate: 478.35 ± 16.14 μm; and ventral: 492.51 ± 16.77 μm, *n* = 51, 44, 42 sections, from 17, 15, 13 animals, respectively; dorsal vs. intermediate *p* = .005, *t* = 2.91, *f* = 1.05, intermediate vs. ventral *p* = .54, *t* = 0.61, *f* = 1.03, dorsal vs. ventral *p* = .03, *t* = 2.23, *f* = 1.02).

**FIGURE 6:**
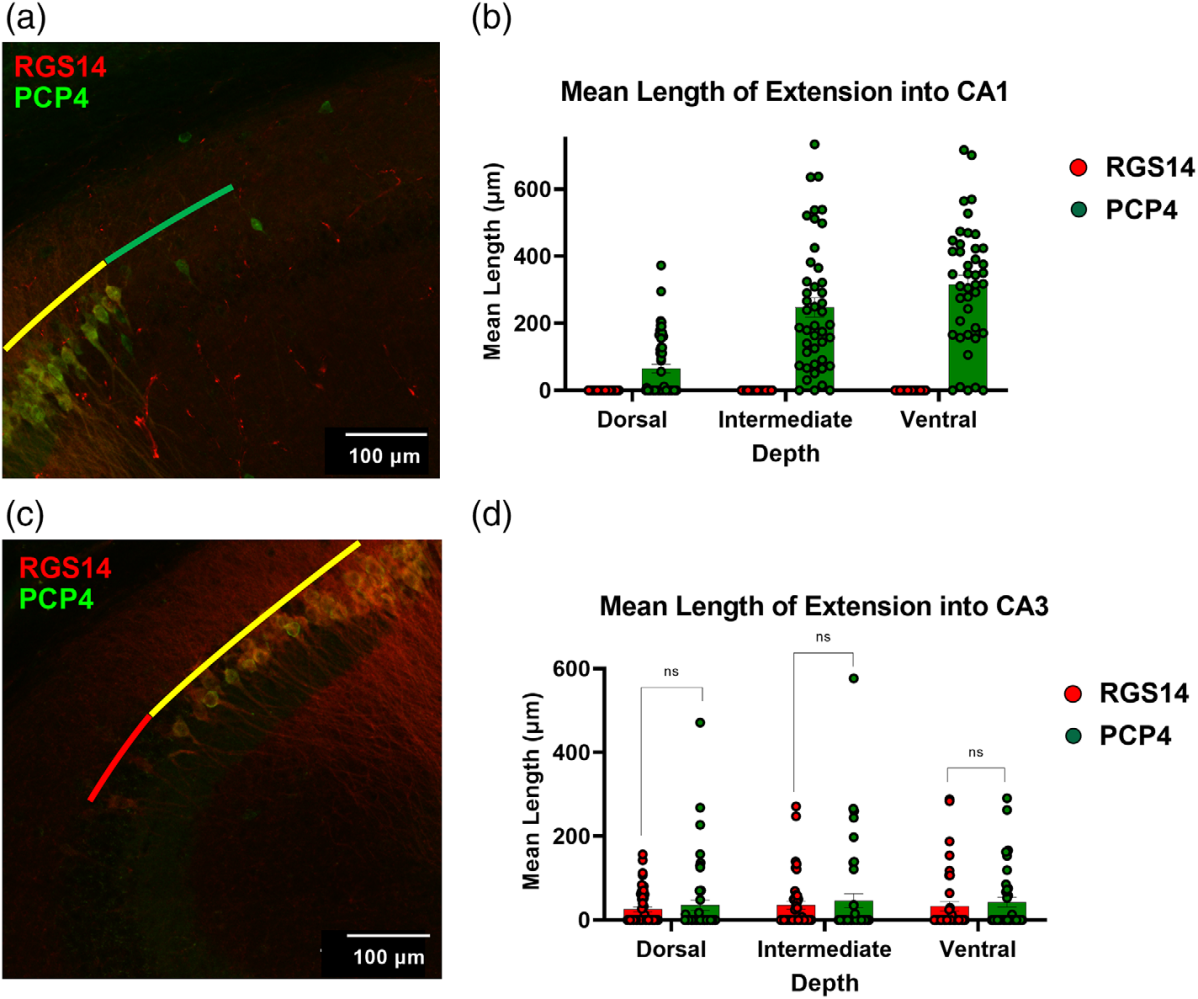
RGS14 and PCP4 staining extends differently beyond their region of overlap. (a) Z‐projected confocal image showing RGS14 and PCP4 immunofluorescence in dorsal hippocampus, including PCP4^+^/RGS14^−^ neuronal labeling (green) beyond the CA1 border of the RGS14^+^/PCP4^+^ double‐labeled zone (DLZ; yellow). (b) Mean lengths of RGS14^+^/PCP4^−^ and PCP4+/RGS14− labeling beyond the DLZ and into CA1. PCP4^+^/RGS14^−^ neuronal labeling beyond the DLZ CA1 border is most striking in ventral depths (ventral: 315.47 ± 27.78 μm; intermediate: 247.72 ± 29.02 μm; dorsal: 65.06 ± 12.937 μm. *n* = 42, 44, 59 sections, from 13, 15, 17 animals). (c) Z‐projected confocal image showing RGS14^+^/PCP4^−^ neuronal labeling (red) beyond the CA3 border of the DLZ (yellow). (d) Mean lengths of RGS14^+^/PCP4^−^ labeling and PCP4^+^/RGS14^−^ labeling beyond the proximal/CA3 border of the DLZ are similar along the dorsoventral axis (dorsal: RGS14^+^ 26.00 ± 5.75 vs. PCP4^+^ 35.39 ± 12.13 μm, *n* = 51 slices, 17 animals; intermediate: RGS14^+^ 35.22 ± 9.56 μm vs. PCP4^+^ 46.11 ± 16.77 μm, *n* = 44 slices, 15 animals; ventral: RGS14^+^ 32.76 ± 11.30 μm vs. PCP4^+^ 42.95 ± 11.50 μm, *n* = 42 slices, from 13 animals).

Research focusing on the functional role of hippocampal area CA2 has been aided by numerous immunohistochemical markers of proteins that exhibit specific enrichment in CA2 neurons. Given our finding that the extent of RGS14 labeling in the hippocampus is dependent on the position within the dorsal–ventral axis, we next sought to determine (1) the degree to which RGS14 labeling colocalizes with another hallmark label of CA2, PCP4, and (2) whether that colocalization is dependent on dorsal–ventral location. For these experiments, we performed IHC to label both RGS14 and PCP4 in dorsal, intermediate, and ventral horizontal sections (Supplementary S1) from adult wild‐type mice (Figure 5a–c). Consistent with our previous results, we observed that the proximodistal extent of RGS14 labeling decreased along the dorsal‐intermediate‐ventral axis. The mean length of RGS14 labeling was significantly greater in dorsal sections when compared with both intermediate and ventral positions (Figure 5d, dorsal: 571.25 ± 13.58 μm vs. intermediate; 516.56 ± 13.29 μm, *p* = .006, dorsal vs. ventral; 526.04 ± 15.75 μm, *p* = .033, *n* = 59, 44, 42 sections from 17, 15, 13 animals, respectively). Conversely, PCP4+ labeling significantly *increased* along the same axis with a mean length of 648.98 ± 21.42 μm in dorsal hippocampus, 774.23 ± 38.44 μm in intermediate sections and 852.96 ± 35.46 μm in ventral sections (Figure 5e: *n* = 51, 44, 42 sections, respectively from 17, 15, 13 animals). Interestingly, the region of overlap, or DLZ, between these two CA2 markers, was significantly greater in dorsal sections compared with intermediate and ventral sections, similar to RGS14+ measures reported in Figure 3e,f (Figure 5f, dorsal: 543.20 ± 15.34 μm, intermediate: 478.35 ± 16.14 μm, and ventral: 492.51 ± 16.77 μm, *n* = 51, 44, 42 sections, from 13 animals). While quantifying this overlap, we observed a pattern wherein these markers labeled neurons beyond the double labeled region, and so we aimed to quantify the extension of these hallmark CA2 markers beyond the DLZ in the direction of neighboring CA1 (Figure 6a, green) and CA3 (Figure 6c, red).

Although we found no evidence of RGS14‐positive cells ranging into area CA1 beyond the RGS14+/PCP4+ DLZ/CA2, we did observe PCP4+/RGS14− neuronal labeling extending beyond the DLZ and into CA1 (Figure 6a and Supplementary S4a,b, see Methods for quantification criteria). Furthermore, this extension was dependent on dorsal–ventral depth with the largest mean length of extension beyond the DLZ seen in ventral sections (315.47 ± 27.78 μm, 93% of sections), followed by intermediate (247.72 ± 29.02 μm, 93% of sections) and dorsal levels (65.06 ± 12.94 μm, 43% of sections; Figure 6a and Supplementary S4c).

Regarding neuronal labeling proximal to the DLZ, towards CA3, we observed instances in which both RGS14+/PCP4‐ and PCP4+/RGS14− “straggler cells” extended beyond the DLZ. We found no significant difference in the mean length of these extensions of either labeled populations at any of the dorsoventral depths measured (Figure 6d, dorsal: RGS14+ 26.00 ± 5.75 μm vs. PCP4+ 35.39 ± 12.13 μm, *n* = 51 sections, from 17 animals; intermediate: RGS14+ 35.22 ± 9.56 vs. PCP4+ 46.11 ± 16.77 μm, *n* = 44 sections, from 15 animals; ventral: RGS14+ 32.76 ± 11.30 μm vs. PCP4+ 42.95 ± 11.50 μm, *n* = 42 sections, rom 13 animals) though, at both levels, we found a higher percentage of quantified sections exhibiting RGS14+ staining beyond the DLZ and into CA3 (Supplementary S4c, dorsal: RGS14+ extension 37% vs. PCP4+ extension 25%, *n* = 51 sections; intermediate: RGS14+ 41% vs. PCP4+ 25%, *n* = 44 sections). In ventral hippocampus, RGS14+/PCP4− straggler cells were less common and observed in only 26% of analyzed sections versus in 36% of sections with PCP4+/RGS14− cells (Supplementary S4d), though we found no significant difference in length of extension at this depth (Figure 6d, ventral: *n* = 42 sections, from 13 animals). Taken together, we find reliable and continuous colocalization of RGS14 and PCP4 labeling across the dorsal–ventral axis, consistent with previous work, but observe heterogeneity among individually labeled neurons that extend beyond this double labeled region. Although RGS14 aligns well with the DLZ at the putative CA2/1 border, PCP4+ neurons were found to extend well into CA1 at all three DV levels and most abundantly at more ventral depths. Conversely, although we found both RGS14+ and PCP4+ neurons extending beyond the CA2/3 end of the DLZ, RGS14 staining was more likely to extend at both dorsal and intermediate depths and PCP4 being more likely to extend into ventral CA3.

### 3.3 PNNs as a marker of CA2

**FIGURE 7:**
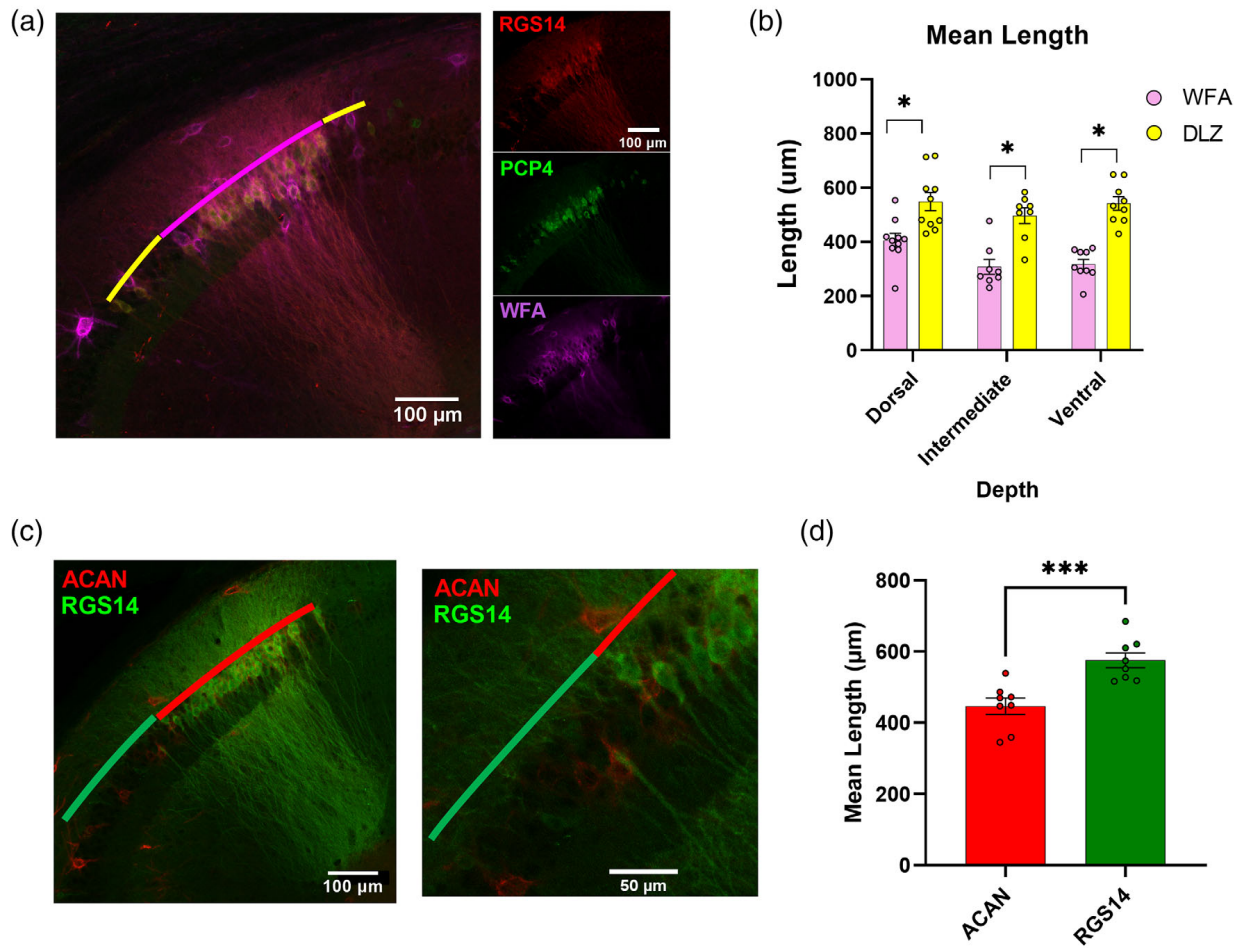
Staining for perineuronal nets does not label the entirety of CA2. (a) Left, Representative image of RGS14, PCP4, and *Wisteria Floribunda* agglutinin (WFA) immunofluorescence (20× magnification). WFA labeling (magenta) does not completely overlap with the distal‐most and proximal‐most ends of the RGS14^+^/PCP4^+^ double‐labeled zone (DLZ; yellow). Right, Confocal images of single stains. (b) Mean length of WFA immunopositivity is significantly shorter than that of the RGS14/PCP4^+^ DLZ throughout the dorsoventral axis. (dorsal WFA = 405.33 ± 26.10 vs. DLZ = 548.15 ± 33.57 μm *p* = .004, *t* = 3.36, *f* = 1.66, *n* = 10 sections from 3 animals; intermediate WFA = 308.34 ± 27.83 μm vs. DLZ = 496.42 ± 28.94 μm *p* = 0004, *t* = 4.68, *f* = 1.08, *n* = 8 sections from 3 animals; ventral WFA = 318.10 ± 18.54 μm vs. DLZ = 541.88 ± 25.06 μm *p* ≤ .000, *t* = 7.18, *f* = 1.83 *n* = 9 sections, from 3 animals). (c1) Representative image of ACAN and RGS14 immunofluorescence. (c2) Expanded view of inset from (c1). (d) Mean length of Aggrecan immunopositivity is significantly shorter than that of RGS14 (ACAN^+^ = 575.44 ± 21.10 μm vs. RGS14^+^ = 446.02 ± 22.76 μm, *p* = .001, *t* = 4.17, 1.66, *n* = 8 dorsal sections, from 3 animals).

PNNs are a chondroitin sulfate‐rich extracellular matrix typically found surrounding inhibitory neurons throughout the brain (Dityatev et al., 2010). Recent research, though, has highlighted the unique association with glutamatergic pyramidal neurons in area CA2 and their role in restricting long‐term potentiation (Carstens et al., 2016). Again applying IHC in horizontal mouse brain sections, we investigated whether the lectin WFA or an antibody against the proteoglycan ACAN, both of which label PNNs (Giamanco et al., 2010), could serve as a singular proxy for the RGS14+/PCP4+ DLZ (Figure 7a,b).

**FIGURE 8:**
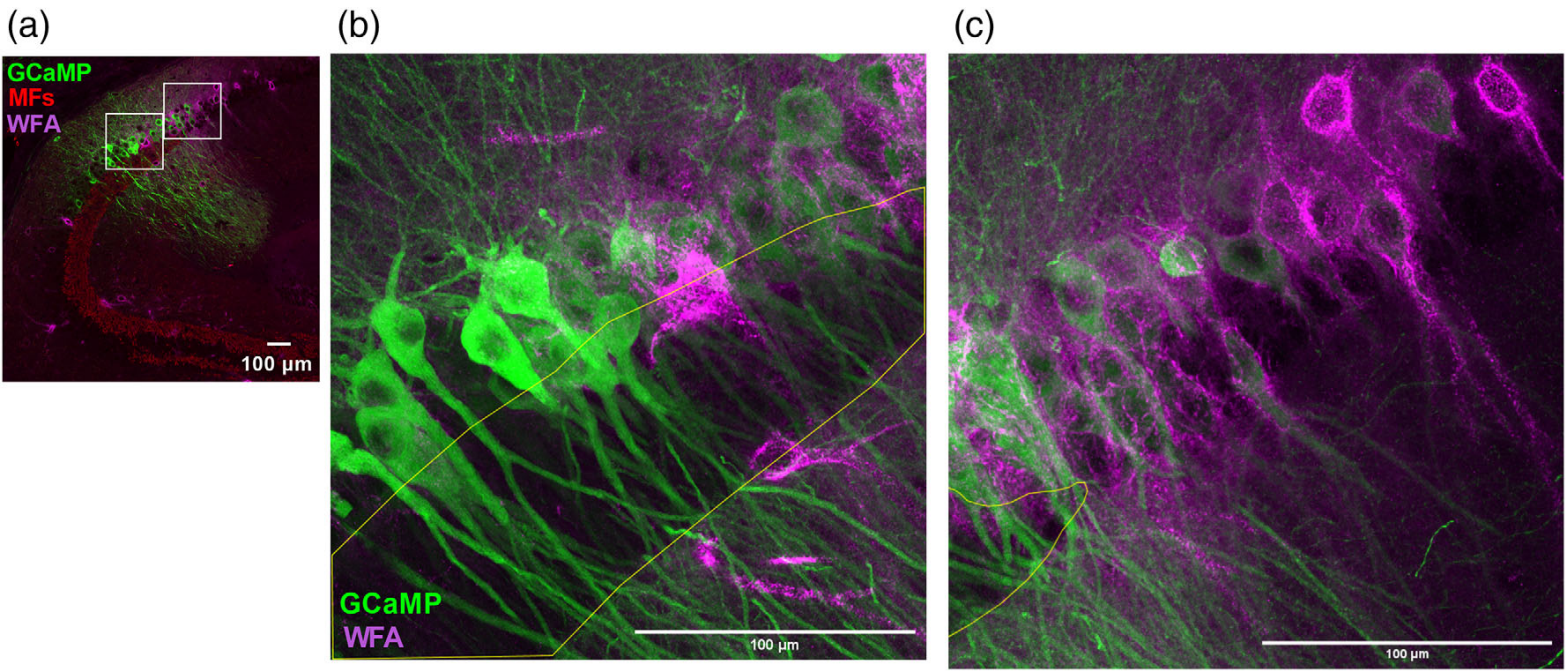
*Wisteria Floribunda* agglutinin (WFA) most strongly associates with mossy fiber (MF)‐negative CA2 neurons. (a) Reference overview image (40×; 1024 × 1024 pixels) of a horizontal section from dorsal hippocampus showing WFA (magenta) and Znt3 (red) immunopositivity and GCaMP6f‐expressing CA2/CA3 pyramidal neurons. Insets are expanded (2048 × 2048 pixels) in (b) and (c). (b,c) Expanded views from (a) showing WFA‐immunopositive perineuronal nets surrounding GCaMP‐expressing CA2 pyramidal neurons, with Znt3‐immunopositive *stratum lucidum* outlined in yellow. Intensity of WFA staining is strongest in the MF‐negative CA2 (c) and markedly reduced towards the CA3/CA2 border (b). All scale bars = 100 μm.

At all depths along the dorsal–ventral axis, the mean length of WFA staining was significantly shorter than the RGS14+/PCP4+ double labeled zone (dorsal: WFA labeling is 26% sorter than DLZ, intermediate: 38%, ventral: 41%). This finding is most pronounced in intermediate and ventral sections (Figure 7b intermediate WFA = 308.34 ± 27.83 μm vs. DLZ = 96.42 ± 28.94 μm, *n* = 8 sections from 3 animals, *p* = .0004; ventral WFA = 318.094 ± 18.538 μm vs. DLZ = 541.878 ± 25.060 μm *p* ≤ .0001, *n* = 9 sections from 3 animals), though the difference was observed in dorsal‐most sections as well (Figure 7b dorsal WFA = 405.33 ± 26.10 μm, DLZ = 548.15 ± 33.57 μm, *p* = .0035, *n* = 10 sections from 3 animals). Furthermore, the mean length of WFA staining is significantly longer in dorsal sections versus intermediate and ventral (dorsal vs. intermediate 405.30 vs. 308.30 μm *p* = .02, *t* = 2.53, *f* = 1.10, *n* = 10, 8 slices from 3 animals; dorsal vs. ventral 405.30 vs. 318.10 μm *p* = .02, *t* = 2.67, *f* = 2.20, *n* = 10, 9 sections from 3 animals). This pattern is not biased to either the proximal (CA3) or the distal (CA1) end of the DLZ (*data not shown*). Given the fact that WFA is also known to label interneurons in the region, cells that were WFA but RGS14−/PCP4− were not included in WFA+ length measurements. Similarly, the mean length of immunopositivity for the PNN component ACAN was also significantly shorter at dorsal depths when compared with RGS14+ (Figure 7d dorsal ACAN+ = 575.44 ± 21.10 μm vs. RGS14+ = 446.02 ± 22.76 μm *p* = .001, *n* = 8 sections from 3 animals). Interestingly, when CA2 neurons were labeled with the fluorescent reporter GCaMP6f in combination with IHC staining of WFA and Znt3 (Figure 8a) and imaged at 40× magnification, we saw that WFA labeling was the strongest in the region just distal to the end of s*tratum lucidum* (Figure 8b, stratum *lucidum* borders outlined in yellow), a region that we showed earlier was RGS14+ (Figure 3b,f) and presumably lacks excitatory input from dentate granule cells. The intensity of WFA labeling drops off steeply from the CA1 towards the proximal, CA3‐associated end of the CA2–CA3 axis (Figure 8a,c). This gradient supports the idea of PNN‐driven heterogeneity in hippocampal area CA2.

### 3.4 STEP and VGluT2 labeling align differently with the proximal and distal ends of the DLZ

**FIGURE 9:**
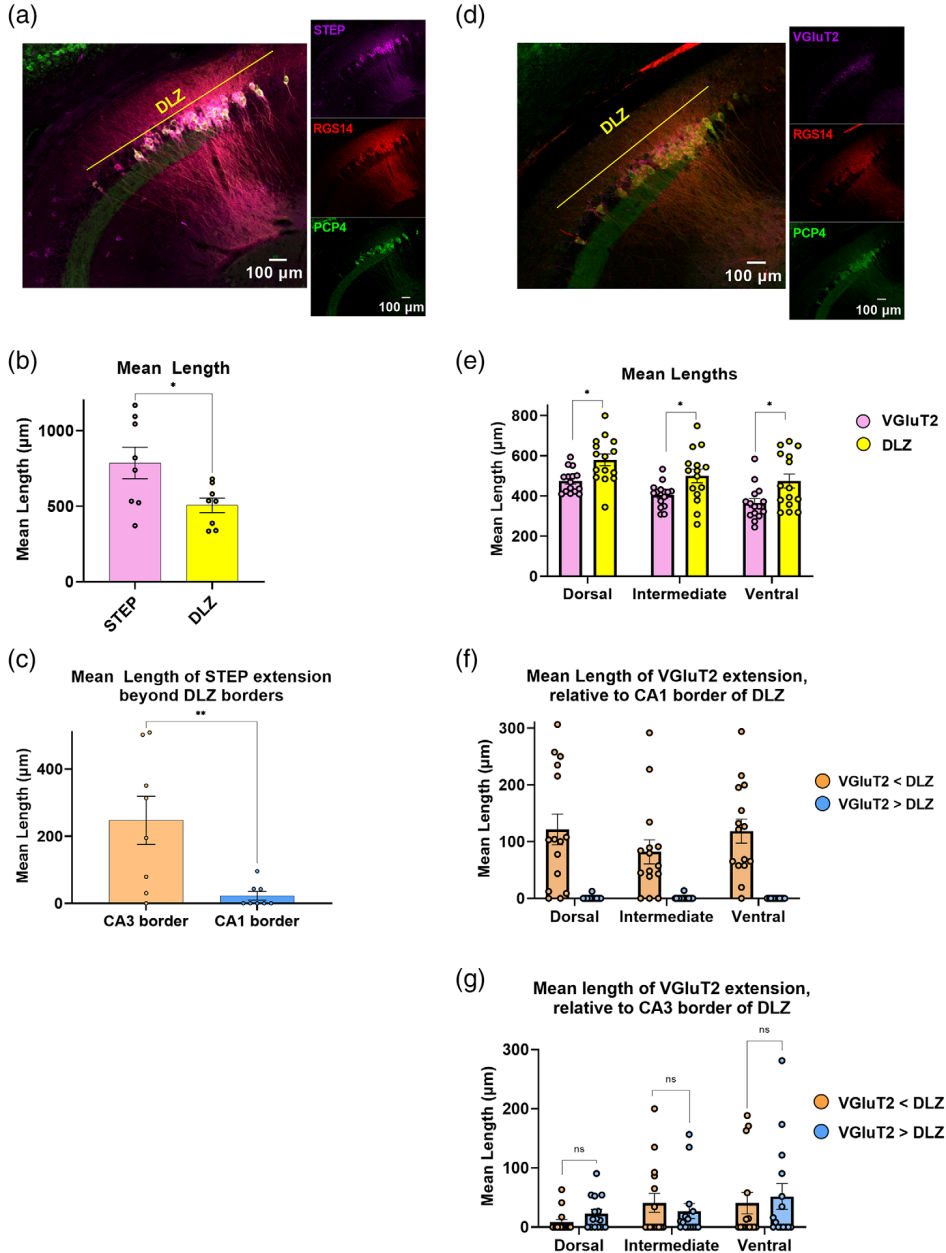
STEP and VGluT2 do not completely overlap with the RGS14+/PCP4+ double‐labeled zone (DLZ). (a) Left, Confocal image showing immunofluorescence of STEP (magenta), RGS14 (red), and PCP4 (green) in horizontal section from dorsal hippocampus. Colored lines indicate RGS14+/PCP4+ DLZ (yellow). Right, Single stain confocal images. (b) In dorsal hippocampus, mean length of the RGS14^+^/PCP4^+^ DLZ is significantly shorter than that of STEP labeling. (STEP = 786.95 ± 104.93 μm vs. DLZ = 506.41 ± 48.45 μm *p* = .03, *t* = 2.43, *f* = 4.69, *n* = 8 dorsal sections total from 2 animals, *n* = 4 sections per animal). (c) Extension of STEP^+^ straggler cells beyond the DLZ are predominant at the CA2/3 border. The mean length of extension beyond the proximal/CA3 border which equals 247.44 ± 71.65 μm versus mean length of extension beyond CA1 border which equals 22.68 ± 12.48 μm Welch's *p* = .036, Welch's corrected *t* = 2.43, *f* = 4.69). (d) Left, Confocal image showing immunofluorescence of VGluT2 (magenta), RGS14 (red), and PCP4 (green). Right, Single stain confocal images. (e) Mean length of dense VGluT2 labeling in the pyramidal layer is significantly shorter than the mean length of DLZ, in all dorsoventral depths (dorsal VGluT2 = 474.26 ± 15.356 μm vs. DLZ = 580.31 ± 0.94 μm Welch's *p* = .004, Welch's corrected *t* = 3.22, *f* = 3.60; intermediate VGluT2 = 404.6 ± 15.75 μm vs. DLZ = 499.4 ± 33.94 μm Welch's *p* = .02, Welch's corrected *t* = 2.54, *f* = 4.64; ventral VGluT2 = 364.41 ± 22.19 μm vs. DLZ = 474.28 ± 34.93 μm *p* = .013, *t* = 2.65, *f* = 2.5, *n* = 45 sections total, 5 sections per dorsoventral depth from 5 animals). (f) VGluT2 expression falls short of the DLZ/CA1 border in nearly all analyzed sections (43 of 45 sections) along the dorsal‐intermediate‐ventral axis (orange bars). Mean length by which VGluT2 staining fall short of the DLZ/CA1 border vs. mean length of VGluT2 staining extending beyond the DLZ/CA1 border (dorsal 121.55 ± 27.12 μm vs. 0.84 ± 0.84 μm; intermediate 82.09 ± 21.23 μm vs. 0.92 ± 0.92 μm; ventral 118.38 ± 21.14 μm vs. 0.00 ± 0.00 μm, *n* = 45 sections total, 5 sections per dorsoventral depth from 5 animals). (g) Mean length by which VGluT2 falls short of the DLZ/CA3 border (orange bars) compared with mean length of VGluT2 staining extending beyond the DLZ CA3 border (blue bars) along the dorsal‐intermediate‐ventral axis. (dorsal 8.14 ± 4.90 μm vs. 22.94 ± 7.20 μm *p* = 0.1, *t* = 1.7, *f* = 2.15; intermediate 40.95 ± 16.14 μm vs. 27.16 ± 12.84 μm *p* = .051, *t* = 0.67, *f* = 1.58; ventral 40.60 ± 18.32 μm vs. 0.00 ± 0.00 μm *p* = .69, *t* = 0.40, *f* = 1.37, *n* = 45 sections total, 5 sections per dorsoventral depth from 5 animals).

The Striatal‐Enriched protein tyrosine Phosphatase (STEP) and the Vesicular Glutamate Transporter 2 (VGluT2) contribute to the distinct molecular profile of the CA2 region and label CA2 soma and SuM presynaptic terminals, respectively (Chen et al., 2020; McCann et al., 2021). We therefore stained horizontal hippocampal sections to investigate whether either STEP or VGluT2 labeling can serve as a single immunohistochemical proxy for the RGS14+/PCP4+ DLZ (Figure 9a,d). Similar to what we observed with RGS14+ staining, STEP labeling extends significantly beyond the DLZ (Figure 9b). This greater mean length is driven predominantly by the extension of STEP+ cells beyond the DLZ and into distal CA3, with STEP labeling aligning tightly with the CA1 border of DLZ/CA2, like RGS14 (Figure 9c). Conversely, the mean length of VGluT2‐positive axon terminal staining is, on average, significantly shorter than the DLZ length in all levels along the dorsal–ventral axis (Figure 9e). However, in contrast to RGS14 and STEP labeling, we found that VGluT2 staining consistently falls short of the DLZ bordering CA1 by ~100 μm in dorsal, intermediate, and ventral sections (Figure 9f, orange bars). Interestingly, we observed either alignment or minimal extension of dense VGluT2+ in the pyramidal cell layer beyond the proximal border of the DLZ and into CA3 in the majority of dorsal and intermediate sections analyzed. This was most striking in dorsal slices where the average mean length by which VGluT2 staining fell short of the DLZ/CA3 border was 8.14 ± 4.90 μm (orange bars, 3 of 15 dorsal sections) while sections that showed VGluT2 extension beyond the DLZ/CA3 border had a mean length of extension of 22.94 ± 7.20 μm (blue bars, 9 of 15 dorsal slices). Of the 15 dorsal sections analyzed, 3 showed alignment with the proximal end of the DLZ, that is, VGluT2 staining neither fell short nor extended beyond the DLZ towards CA3 (mean length of extension beyond CA3: dorsal, 22.94 ± 7.20 μm; intermediate, 27.16 ± 12.84 μm, 45 sections total, 3 sections per dorsoventral depth from 5 animals; Figure 9g). Taken together, both STEP and VGluT2 expression patterns may be useful tools to differentiate CA2 (as defined by the DLZ) from area CA1 and CA3, respectively, along the dorsal–ventral axis. Although STEP shows consistent extension into area CA3 in dorsal sections, VGluT2‐positive fibers can be used to distinguish CA3 from CA2 in more dorsal and intermediate levels, but is less reliable in the ventral hippocampus where extension into CA3 is more prominent. Last, we note that the mean fluorescence intensity of VGluT2 labeling in *SO* is significantly greater in ventral levels than in dorsal levels (Figure S5b) consistent with previous work that observed greater relative length of SuM innervation when comparing dorsal versus ventral hippocampal coronal sections (Chen et al., 2020).

### 3.5 Colocalization of a genetically encoded CA2 marker with the DLZ

**FIGURE 10:**
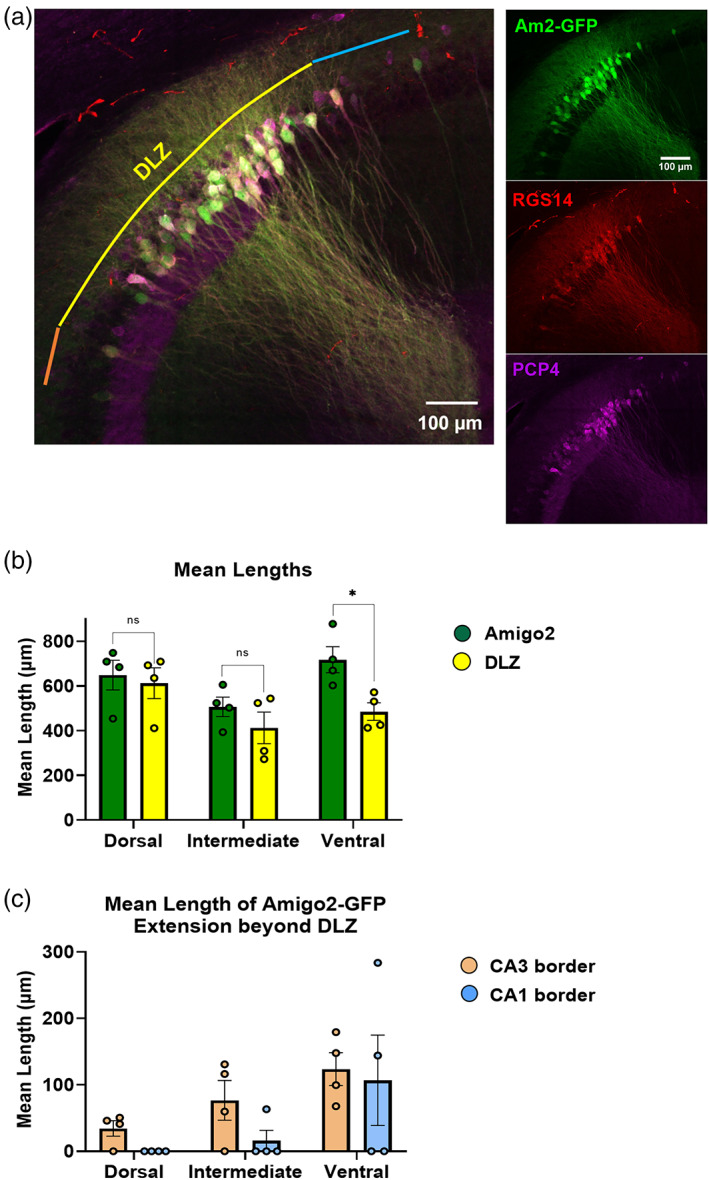
Amigo2‐GFP labeling and double‐labeled zone (DLZ) do not overlap completely along the dorsoventral axis of hippocampus. (a, Left) 20× Confocal image of RGS14 (red) and PCP4 (magenta) immunofluorescence and Amigo2‐GFP expression (green) in a horizontal section from dorsal hippocampus. Colored lines indicate RGS14^+^/PCP4^+^ DLZ (yellow), as well as extension of Amigo2‐GFP labeling beyond the CA3 and CA1 borders (orange and blue, respectively). (Right) Confocal images of individual stains. (b) Mean length of Amigo2‐GFP labeling is statistically similar to that of the RGS14^+^/PCP4^+^ DLZ in dorsal and intermediate hippocampus. (dorsal Am2‐GFP = 649.00 ± 66.35 μm vs. DLZ = 612.15 ± 68.87 μm *p* = .71, *t* = 0.39, *f* = 1.08; intermediate Am2‐GFP = 506.53 ± 43.67 μm vs. DLZ = 412.51 ± 70.66 μm *p* = .30, *t* = 1.13, *f* = 2.62; ventral Am2‐GFP = 717.22 ± 58.61 vs. DLZ = 485.18 ± 39.14 μm *p* = .012, *t* = 3.29, *f* = 2.42, *n* = 4 sections per dorsoventral depth from 2 animals). (c) Along the dorsoventral axis, extension of Amigo2‐GFP labeling beyond the DLZ is predominant at the DLZ/CA3 border (orange bars), as opposed to the DLZ/CA1 border (blue bars) with the exception of ventral sections (dorsal Am2‐GFP CA3 extension = 34.59 ± 11.66 μm vs. CA1 extension = 0.00 ± 0.00 μm *p* = .03, *t* = 2.96, *f* = n/a; intermediate Am2‐GFP CA3 extension = 76.75 ± 29.82 μm vs. CA1 extension = 15.83 ± 15.83 μm *p* = .12, *t* = 1.80, *f* = 3.55; ventral Am2‐GFP CA3 extension = 123.69 ± 24.79 vs. CA1 extension = 106.94 ± 68.00 μm *p* = .82, *t* = 0.23, *f* = 7.52, *n* = 4 sections per dorsoventral animals from 2 animals).

The CA2‐specific enrichment of the cell‐adhesion protein Amphoterin‐induced gene and open reading frame 2 (AMIGO2) has been employed by many labs as a genetically encoded marker that can be used to not only distinguish CA2 from CA1 and CA3 but also to manipulate CA2 neuronal activity in vivo during specific behavioral tasks (Alexander et al., 2019; Hitti & Siegelbaum, 2014; Laeremans et al., 2013; Leroy et al., 2018; Meira et al., 2018). To investigate how genetically encoded GFP under the control of the *Amigo2* promoter aligns with the DLZ, we performed IHC using RGS14 and PCP4 antibodies on horizontal hippocampal sections from Amigo2‐GFP mice (Figure 10a). Although the mean length of GFP expression and the DLZ are statistically similar in dorsal and intermediate hippocampus (dorsal Am2‐GFP = 649.00 ± 66.35 μm vs. DLZ = 612.15 ± 68.87 μm, *p* = 0.71; intermediate Am2‐GFP = 506.53 ± 43.67 vs. DLZ = 412.508 ± 70.66, *p* = 0.30), the mean length of Amigo2‐GFP labeling is significantly longer than that of the DLZ in ventral levels (Am2‐GFP = 717.21 ± 58.61 vs. DLZ = 485.18 ± 39.14, *p* = .012, *n* = 4 sections from 2 animals; Figure 10b). Moreover, we observed extension of GFP labeling beyond the DLZ throughout the dorsoventral axis (Figure 10c). As with many of our immunohistochemical markers like RGS14 and STEP, GFP labeling aligns well with the CA1 border of the DLZ in dorsal and intermediate sections (mean length of extension: dorsal 0.0 ± 0.0 μm; intermediate 15.83 ± 15.83, *n* = 4, 4 sections, respectively) but extends beyond the DLZ in more ventral sections (mean length of extension: ventral 106.94 ± 67.98, *n* = 4 sections). With respect to the CA2/3 border region, GFP positive neurons extending beyond the CA3 border of DLZ are observed at all levels and increase in length along the dorsal‐intermediate‐ventral axis (mean length of extension: dorsal 34.59 ± 11.66 μm; intermediate 76.75 ± 29.82 μm; ventral 123.69 ± 24.79 μm, *n* = 4, 4, 4 sections, respectively from 2 animals; Figure 10c). Although we did see sparse examples of GFP+ positive neurons extending beyond DLZ that were immuno‐negative for both RGS14 and PCP4, it was more common for GFP+ stragglers to be PCP4+/RGS14− (*data not shown*). This was especially true with respect to PCP4 extension into CA1, which as we have shown, increases in likelihood in more intermediate and ventral hippocampal sections.

## 4 DISCUSSION

Here, we sought to characterize common morphological and immunohistochemical indicators of area CA2 in mouse hippocampal sections given that this region, while smaller in relation to CA3 and CA1, is a functionally important part of the hippocampal circuit. Moreover, given the increasing complexity in experiments that often require multiple fluorescent markers that fill the available spectral range, we thought it valuable to compare and contrast commonly used CA2 markers to determine if labeled populations are comparable and, if not, to better understand how different molecular markers may be better suited than others for a given hypothesis.

Consistent with previous work (Kohara et al., 2014), we observed that both the average MFB area and the total number of boutons are proportional to distance from the distal most end of the MF pathway. Although the number of small (<2 μm^2^) and medium (≥2 μm^2^ < 4 μm^2^) boutons remains relatively consistent along the CA2 → CA3 disto‐proximal axis we observe large MFBs becoming the predominate fraction beginning in the bin 200–300 μm from dSL and continuing through CA3 proper in more dorsal hippocampal sections. We also report that RGS14‐positive neurons extend, on average, ~300 μm from the end of *stratum lucidum* towards area CA3. Therefore, it is possible that the large MFBs that we observe in the 200 300 μm bin form synapses onto CA2 neurons near the CA2/CA3 interface. This idea that MF synapses in CA2 can in fact be comprised of presynaptic and postsynaptic compartments with morphologies similar to that of the much‐studied CA3 synapses is further supported by high resolution, high magnification confocal images that clearly show large, complex postsynaptic spines in *stratum lucidum* at distances <200 μm from the distal most end of the MF pathway (Figures 4b and S2). As expected, these complex spines are absent in neurons that sit beyond the dSL (towards CA1) but within the extent of RGS14+labeling (CA2) which, on average, extends ~170 μm past the end of *stratum lucidum* in dorsal hippocampal sections (Figure 3b,f). Further research will be needed to determine the potential functional differences between mossy fiber positive and MF negative neurons, as well as the role that select large, complex spines may be playing in CA2‐MF synaptic transmission, but as yet, we have not identified any antibody markers that would distinguish such neurons. Though most CA2 neurons did not have the complex spines/TEs characteristic of CA3 neurons, they were present, and as such, the presence or absence of complex spines might not be the most accurate way to distinguish between CA3 and CA2 neurons at the single‐cell level. For our initial morphological characterization of these synapses we opted for non‐Cre dependent viral labeling to visualize MFBs and CA2/3 pyramidal neurons using neuronal position relative the distal end (towards CA1) of *stratum lucidum* as an informed estimate of the CA region we were analyzing. Future work will incorporate cell‐type‐specific labeling of either CA3 neurons (*Grik‐4*) or CA2 neurons (*Amigo‐2*) in concert with immunohistochemical CA2 markers to build upon our reported characterization of complex spines along the apical dendrites of hippocampal pyramidal neurons.

Another benefit of using intercranial injections of fluorescent markers to label pre‐ and post‐ synaptic structures along the MF pathway is the ability to clearly identify the ventrolateral targeting of CA2 neurons just ventral to the granule cell bodies from which the axons originate. The “downward, lateral” turn of the MF axons is specific to area CA2 as MF‐positive staining is largely missing in CA3 caudal to the injection site (Figure 2c). Somewhat surprisingly though, we observed no significant difference in either bouton number or area when compared with comparable bins in more dorsal sections closer to site of injection though both metrics exhibited an increasing trend. Future work may uncover a functional role for this interesting axonal deviation.

Immunohistochemical markers are valuable tools to label area CA2 in ex vivo and in vitro preparations but how consistently do they label the same population of neurons and how do these patterns persist along the dorsal–ventral hippocampal axis? To answer these questions, we compared the proximodistal lengths of commonly used molecular markers of CA2 to the region of RGS14+ and PCP4+ colocalization (DLZ) along the dorsal–ventral axis to gain insight into potentially useful patterns of expression. Throughout our analysis, RGS14 and STEP reliably labeled the distal end of the DLZ with little to no evidence of extension into CA1. PCP4+ staining, on the other hand, consistently extended beyond the DLZ and into CA1 with this observed pattern increasing in scale along the DV axis. Conversely, WFA, ACAN, and VGluT2 positive staining fell significantly short of the distal DLZ at all hippocampal depths. Taken together, PCP4, WFA, ACAN, and VGluT2 exhibit legitimate caveats if being used to confirm the border between areas CA2 and CA1. On the more proximal end of the DLZ, our analysis shows that VGlut2+ positive staining is useful for distinguishing the CA2/CA3 border in dorsal and intermediate sections but is less reliable in more ventral sections. Conversely, staining for STEP extended beyond proximal DLZ and into CA3 with a mean extension length of 247.4 ± 72 μm, while WFA staining mean length fell short of the proximal DLZ border at all measured depths. RGS14 and PCP4 exhibit more variability at the CA2/3 border with respect to position along the DV axis. Though both markers showed similar mean lengths of extension beyond the proximal DLZ, though in separate cells, PCP4 extension was observed in a lower percentage of sections when compared with RGS14 in dorsal and intermediate levels, while the opposite pattern was observed in ventral levels where RGS14 was less likely to extend into CA3 relative to PCP4. We also report that when using an Am2‐GFP mouse line to label CA2 neurons, we observed GFP signal extending beyond the DLZ. Furthermore, these GFP+ neurons were most commonly PCP4+/RGS14− though, in a few instances, we observed GFP+ neurons that were negative for both RGS14 and PCP4. While this may be an interesting finding, we note that, more likely, antibody penetration into the tissue is the limiting factor when comparing IHC staining with the expression of a genetically expressed marker, which will be seen throughout the tissue thickness. Further work would be necessary to confirm any lack of IHC‐derived signal in GFP+ neurons as an indicator of AMIGO2 (Laeremans et al., 2013). Here, we have highlighted specific molecular markers that can be used to reliably distinguish the relative borders of area CA2 in dorsal hippocampus, with double staining for RGS14 and PCP4 being the most reliable way to validate the location of CA2 in more intermediate and ventral sections, specifically at CA2/3 interface. While we observed no evidence of RGS14+/PCP4− or PCP4+/RGS14− in the DLZ, we also recognize that the density of neurons in *stratum pyramidale* can make it difficult to resolve such occurrences. Using a higher magnification may remedy this fact though more likely, the interrogation of molecular heterogeneity at the single‐cell level would be better achieved by molecular techniques like single‐cell polymerase chain reaction (PCR). Future work will also continue the characterization of immunohistochemical markers like STEP and ACAN to better understand how these markers align with the DLZ in more intermediate and ventral hippocampal sections.

Last, while important research has highlighted the unique way in which PNNs in area CA2 associate with pyramidal neurons and contribute to the lack of LTP at CA2 synapses (Carstens et al., 2016; McCann et al., 2021) our analyses indicate that specific immunohistochemical markers of PNNs, specifically WFA and ACAN, alone are unsuitable for complete labeling of area CA2 as defined by the DLZ. Not only do these markers underrepresent the proximodistal span of CA2 (Figure 7b,d), they also show a noticeable gradient wherein WFA staining was intense near the CA2/1 border and became significantly dimmer in more proximal CA2, closer to CA3 (Figure 8b,c). Using cell‐type specific viral labeling of CA2 pyramidal neurons and immunohistochemical labeling of PNNs future work will be required to better quantify WFA+ heterogeneity throughout area CA2 and, moreover, whether or not the presence of complex spines in CA2 apical dendrites preferentially align with WFA− pyramidal neurons. Furthermore, with one marker, it was difficult to distinguish between the PNNs that surround inhibitory neurons in this region from those surrounding nearby pyramidal cells. Including a GAD counterstain to label interneurons in the region would inform future studies focusing on PNN heterogeneity in area CA2. We should note that our work was conducted exclusively using horizontal sections to preserve both MF axons as well as the SL associated, apical dendrites of CA2 pyramidal neurons (Bischofberger et al., 2006). Further work will be required to determine whether the expression patterns we have outlined here are similar in coronal sections and will help to further our understanding of the molecular heterogeneity in the areas immediately adjacent to hippocampal area CA2.

## Supporting information


**FIGURE S1:** Dorsoventral depths of horizontal sections. (a) Lateral view of hippocampus indicating dorsal, intermediate, and ventral depths for analysis. (a1–3) DAPI‐stained horizontal sections showing dentate gyrus anatomies of serial sections corresponding to dorsal (1), intermediate (2), and ventral (3) depths in (a). Dorsoventral depths correspond approximately to interaural depths (3.64, 3.12, and 2.2 mm) of Paxinos and Franklin's horizontal mouse brain atlas (2012).

**FIGURE S2:** Mossy fiber bouton (MFB) increase in number but not mean density along the distoproximal CA2 → CA3 axis. (a1) Reference overview image (40× magnification; 1024 × 1024 pixels) of a dorsal horizontal section showing GCaMP6f‐expressing CA2/CA3 pyramidal neurons and mCherry‐expressing mossy fibers (MFs) originating from dorsal dentate gyrus. (a2–3) Expanded views (40×; 2048 × 2048 pixels) of insets from (a, Left) showing hand‐drawn boundaries of isolated, well‐defined boutons in the distal‐most 0–100 μm of SL (a, *middle*) and 500–600 μm from dSL (a, right). Inset in (a, right) shows example of a typical MFB in CA3. Scale bar = 3.5 μm (b) Total number of MFBs increases along the CA2 → CA3 axis of dorsal hippocampus (*n* = 6 dorsal sections, *n* = 3 mice). (c) Total MFB density in the 0–100 μm bin (relative to the end of *stratum lucidum* in CA2) is statistically similar in CA2 and CA3 (0–100 μm bin [CA2], 0.0042 ± 0.0012 MFBs/μm^2^ vs. 500–600 μm bin [CA3], 0.0053 ± 0.0010 MFBs/μm^2^ *p* = .5168, *n* = 6 dorsal sections from 3 mice).

**FIGURE S3:** Some CA2 pyramidal neurons display thorny excrescences on apical dendrites in *stratum lucidum*. (a,b) Z‐projected confocal images (40×; 2048 × 2048 pixels) of horizontal sections from dorsal hippocampus showing GCaMP6f‐expressing CA2 pyramidal neurons and mCherry‐expressing mossy fiber boutons originating from dorsal dentate gyrus granule cells. Apical dendrites of select CA2 pyramidal neurons display thorny excrescences (white arrows) located within 160 μm (a) and 90 μm (b) from dSL, respectively. Yellow asterisks indicate apical dendrites that lack thorny excrescences. Inset: Enlarged view showing thorny excrescences of a MF‐CA2 synapse.

**FIGURE S4:** Purkinje Cell Protein 4 (PCP4^+^)/Regulator of G‐protein Signaling 14 (RGS14^−^) extension beyond CA1 border of double‐labeled zone (DLZ) is most common in ventral levels. (a,b) Z‐projected confocal images showing PCP4 neuronal labeling in CA1 of horizontal sections from dorsal (a) and ventral (b) hippocampus. (c) Percentage of sections exhibiting PCP4^+^/RGS14^−^ labeling beyond DLZ and into CA1 is largest in ventral hippocampus (dorsal 43%, *n* = 51 sections; intermediate, 93%, 44 sections; ventral, 93%, 42 sections from 17, 15, 13 animals, respectively). (d) In the dorsal and intermediate hippocampus, the percentage of sections that exhibit RGS14^+^/PCP4^−^ labeling beyond the DLZ, into CA3, is larger than the percentage of sections that exhibit PCP4^+^/RGS14^−^ labeling in CA3. RGS14^+^/PCP4^−^ straggler cells in CA3 are rare in the ventral hippocampus (dorsal: RGS14^+^ 25.96 ± 5.75 μm vs. PCP4^+^ 35.39 ± 12.13 μm, *n* = 51 sections; intermediate: RGS14^+^ 35.22 ± 9.56 μm vs. PCP4^+^ 46.11 ± 16.77 μm, *n* = 44 sections; ventral: RGS14^+^ 32.761 ± 11.301 μm vs. PCP4^+^ 42.95 ± 11.50 μm, *n* = 42 sections).

**FIGURE S5:** Dorsoventral differences in the labeling pattern of VGluT2. (a) VGluT2 labeling in the *stratum oriens* extends diffusely into proximal CA3 in ventral (right), but not dorsal (left) depths. (b) The mean fluorescence intensity of VGluT2 labeling in *stratum oriens* is greater in ventral levels (*bottom* right; dorsal 0.00025 ± 0.00002 vs. intermediate 0.00032 ± 0.00002 *p* = .02, *t* = 2.46, *f* = 1.55; intermediate 0.00032 ± 0.00002 vs. ventral 0.00037 ± 0.00002 *p* = .11, *t* = 1.63, *f* = 1.83; dorsal 0.00025 ± 0.00002 vs. ventral 0.00037 ± 0.00002 *p* = .001, *t* = 3.72, *f* = 1.18, *n* = 44 sections total, 4–5 sections per dorsoventral depth from 5 animals). Scale bars = 100 μm.

## Data Availability

The data that support the findings of this study are available from the corresponding author upon reasonable request.
