# Supplementary figures and images for: Morphological and molecular markers of mouse area CA2 along the proximodistal and dorsoventral hippocampal axes
Source: Hippocampus. 2023 Feb 10;33(3):133–49. doi: 10.1002/hipo.23509 (PMC10443601; doi:10.1002/hipo.23509)

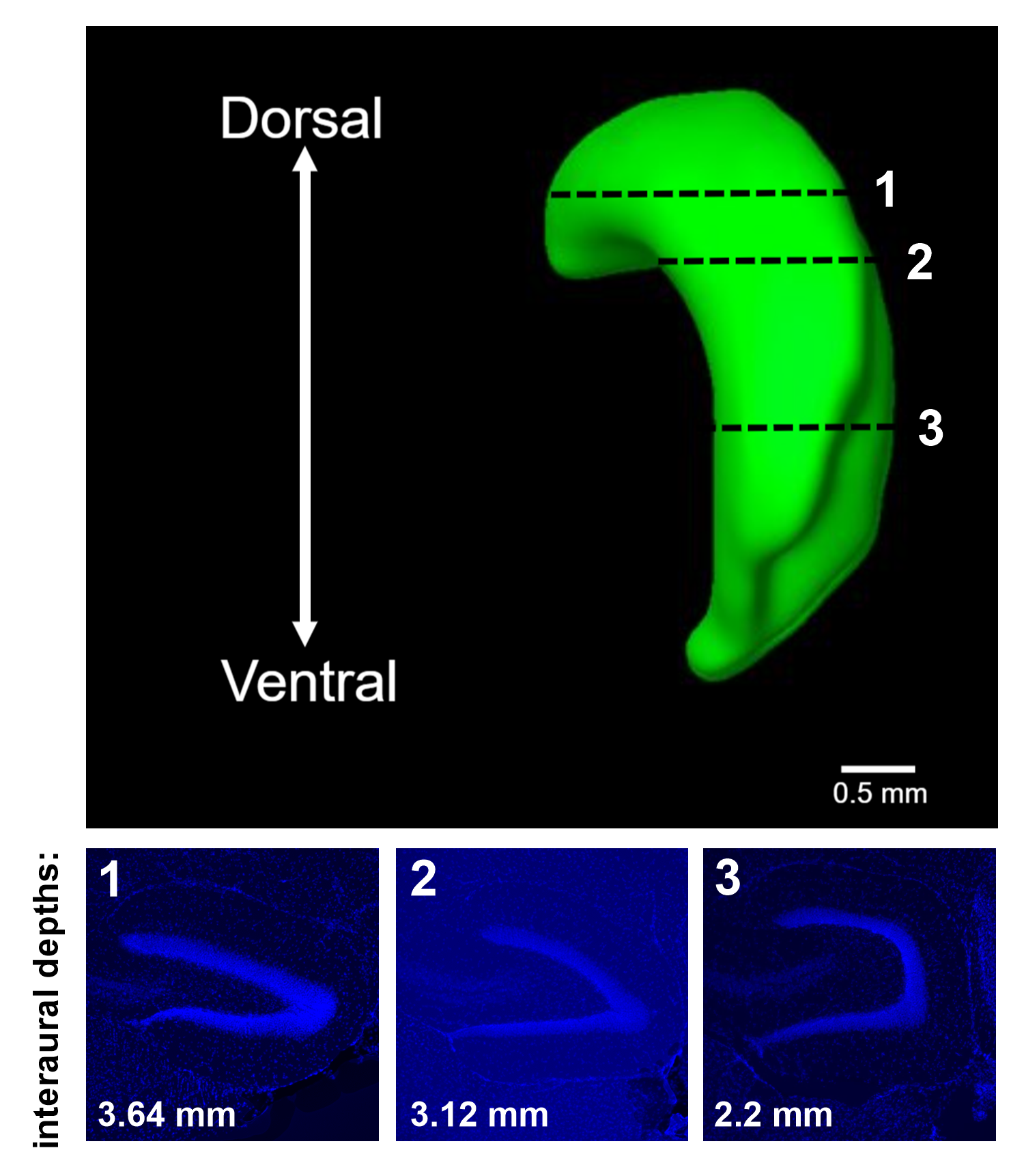

Supplement: Supplementary file 1 — FIGURE S1: Dorsoventral depths of horizontal sections. (a) Lateral view of hippocampus indicating dorsal, intermediate, and ventral depths for analysis. (a1–3) DAPI‐stained horizontal sections showing dentate gyrus anatomies of serial sections corresponding to dorsal (1), intermediate (2), and ventral (3) depths in (a). Dorsoventral depths correspond approximately to interaural depths (3.64, 3.12, and 2.2 mm) of Paxinos and Franklin's horizontal mouse brain atlas (2012). [file HIPO-33-133-s004.tif]

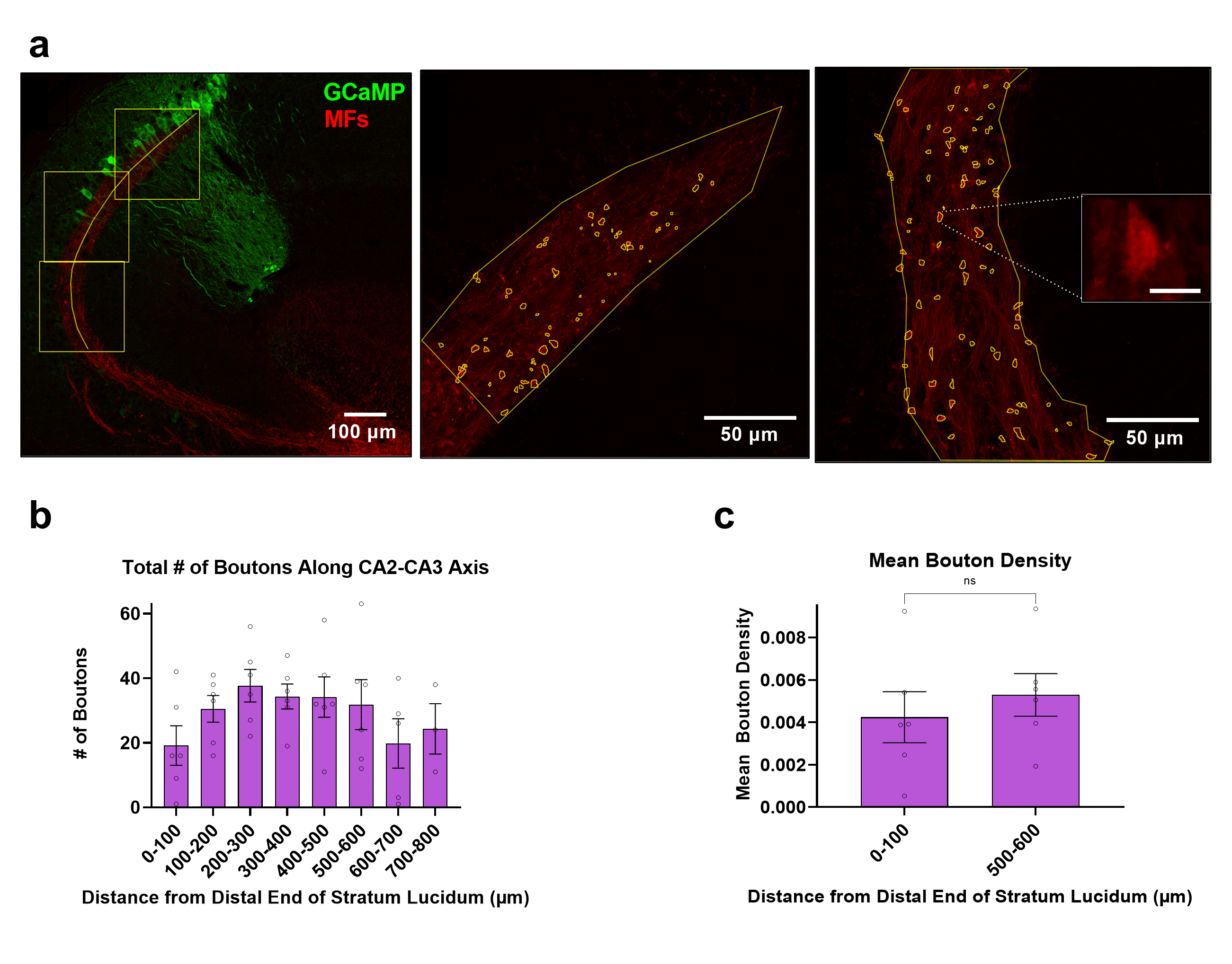

Supplement: Supplementary file 2 — FIGURE S2: Mossy fiber bouton (MFB) increase in number but not mean density along the distoproximal CA2 → CA3 axis. (a1) Reference overview image (40× magnification; 1024 × 1024 pixels) of a dorsal horizontal section showing GCaMP6f‐expressing CA2/CA3 pyramidal neurons and mCherry‐expressing mossy fibers (MFs) originating from dorsal dentate gyrus. (a2–3) Expanded views (40×; 2048 × 2048 pixels) of insets from (a, Left) showing hand‐drawn boundaries of isolated, well‐defined boutons in the distal‐most 0–100 μm of SL (a, middle) and 500–600 μm from dSL (a, right). Inset in (a, right) shows example of a typical MFB in CA3. Scale bar = 3.5 μm (b) Total number of MFBs increases along the CA2 → CA3 axis of dorsal hippocampus (n = 6 dorsal sections, n = 3 mice). (c) Total MFB density in the 0–100 μm bin (relative to the end of stratum lucidum in CA2) is statistically similar in CA2 and CA3 (0–100 μm bin [CA2], 0.0042 ± 0.0012 MFBs/μm2 vs. 500–600 μm bin [CA3], 0.0053 ± 0.0010 MFBs/μm2 p = .5168, n = 6 dorsal sections from 3 mice). [file HIPO-33-133-s003.tif]

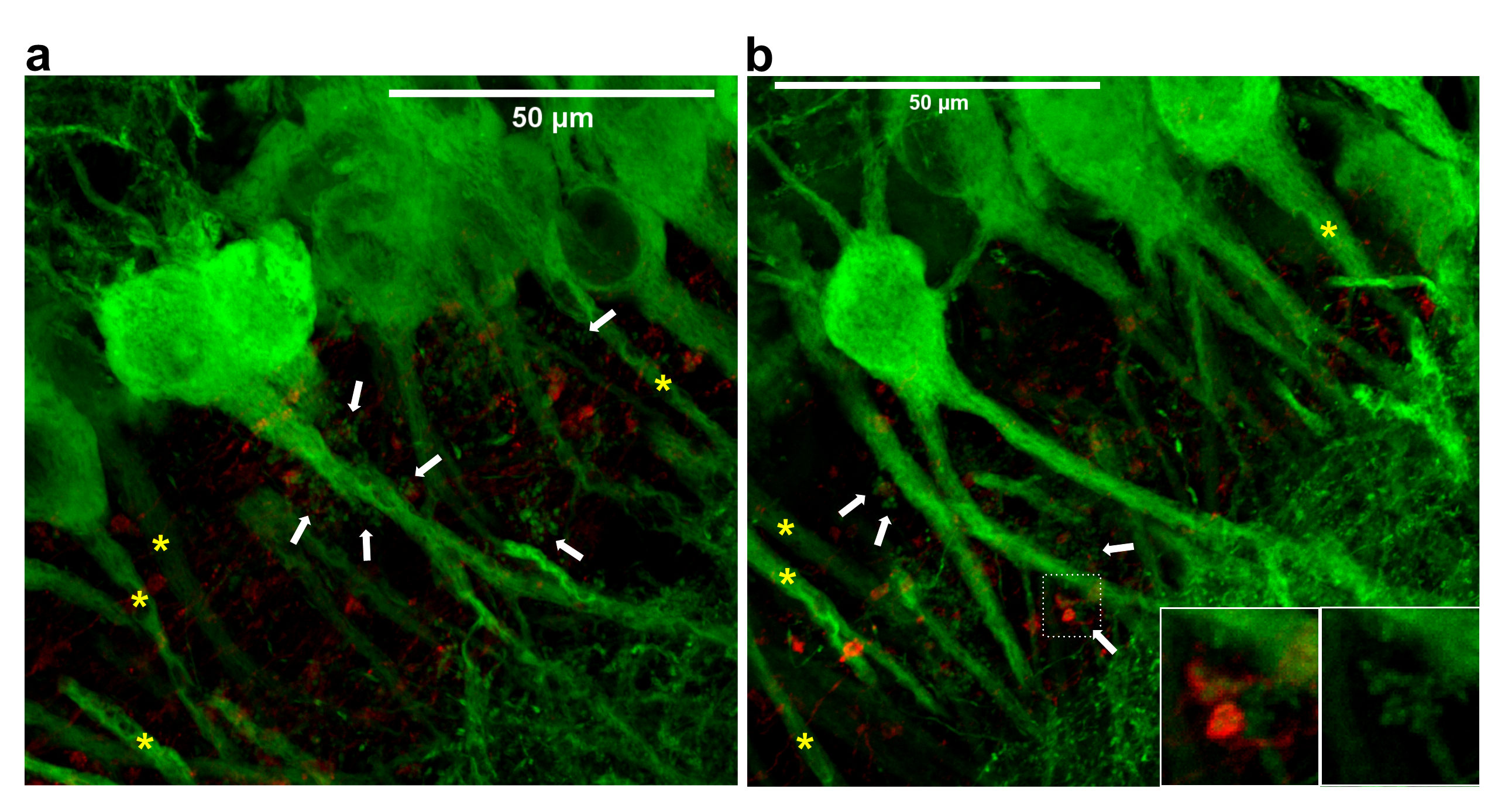

Supplement: Supplementary file 3 — FIGURE S3: Some CA2 pyramidal neurons display thorny excrescences on apical dendrites in stratum lucidum. (a,b) Z‐projected confocal images (40×; 2048 × 2048 pixels) of horizontal sections from dorsal hippocampus showing GCaMP6f‐expressing CA2 pyramidal neurons and mCherry‐expressing mossy fiber boutons originating from dorsal dentate gyrus granule cells. Apical dendrites of select CA2 pyramidal neurons display thorny excrescences (white arrows) located within 160 μm (a) and 90 μm (b) from dSL, respectively. Yellow asterisks indicate apical dendrites that lack thorny excrescences. Inset: Enlarged view showing thorny excrescences of a MF‐CA2 synapse. [file HIPO-33-133-s002.tif]

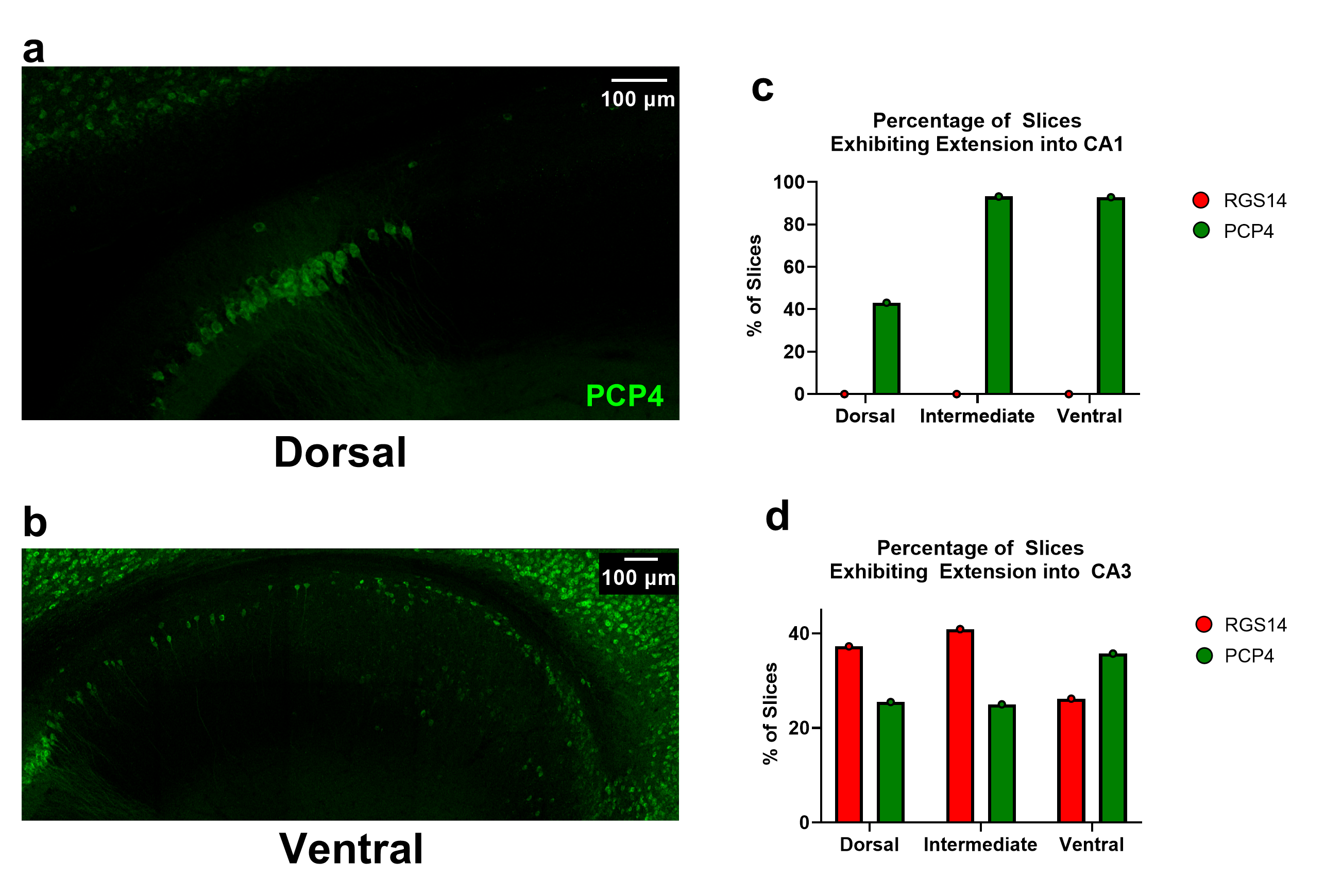

Supplement: Supplementary file 4 — FIGURE S4: Purkinje Cell Protein 4 (PCP4+)/Regulator of G‐protein Signaling 14 (RGS14−) extension beyond CA1 border of double‐labeled zone (DLZ) is most common in ventral levels. (a,b) Z‐projected confocal images showing PCP4 neuronal labeling in CA1 of horizontal sections from dorsal (a) and ventral (b) hippocampus. (c) Percentage of sections exhibiting PCP4+/RGS14− labeling beyond DLZ and into CA1 is largest in ventral hippocampus (dorsal 43%, n = 51 sections; intermediate, 93%, 44 sections; ventral, 93%, 42 sections from 17, 15, 13 animals, respectively). (d) In the dorsal and intermediate hippocampus, the percentage of sections that exhibit RGS14+/PCP4− labeling beyond the DLZ, into CA3, is larger than the percentage of sections that exhibit PCP4+/RGS14− labeling in CA3. RGS14+/PCP4− straggler cells in CA3 are rare in the ventral hippocampus (dorsal: RGS14+ 25.96 ± 5.75 μm vs. PCP4+ 35.39 ± 12.13 μm, n = 51 sections; intermediate: RGS14+ 35.22 ± 9.56 μm vs. PCP4+ 46.11 ± 16.77 μm, n = 44 sections; ventral: RGS14+ 32.761 ± 11.301 μm vs. PCP4+ 42.95 ± 11.50 μm, n = 42 sections). [file HIPO-33-133-s001.tif]

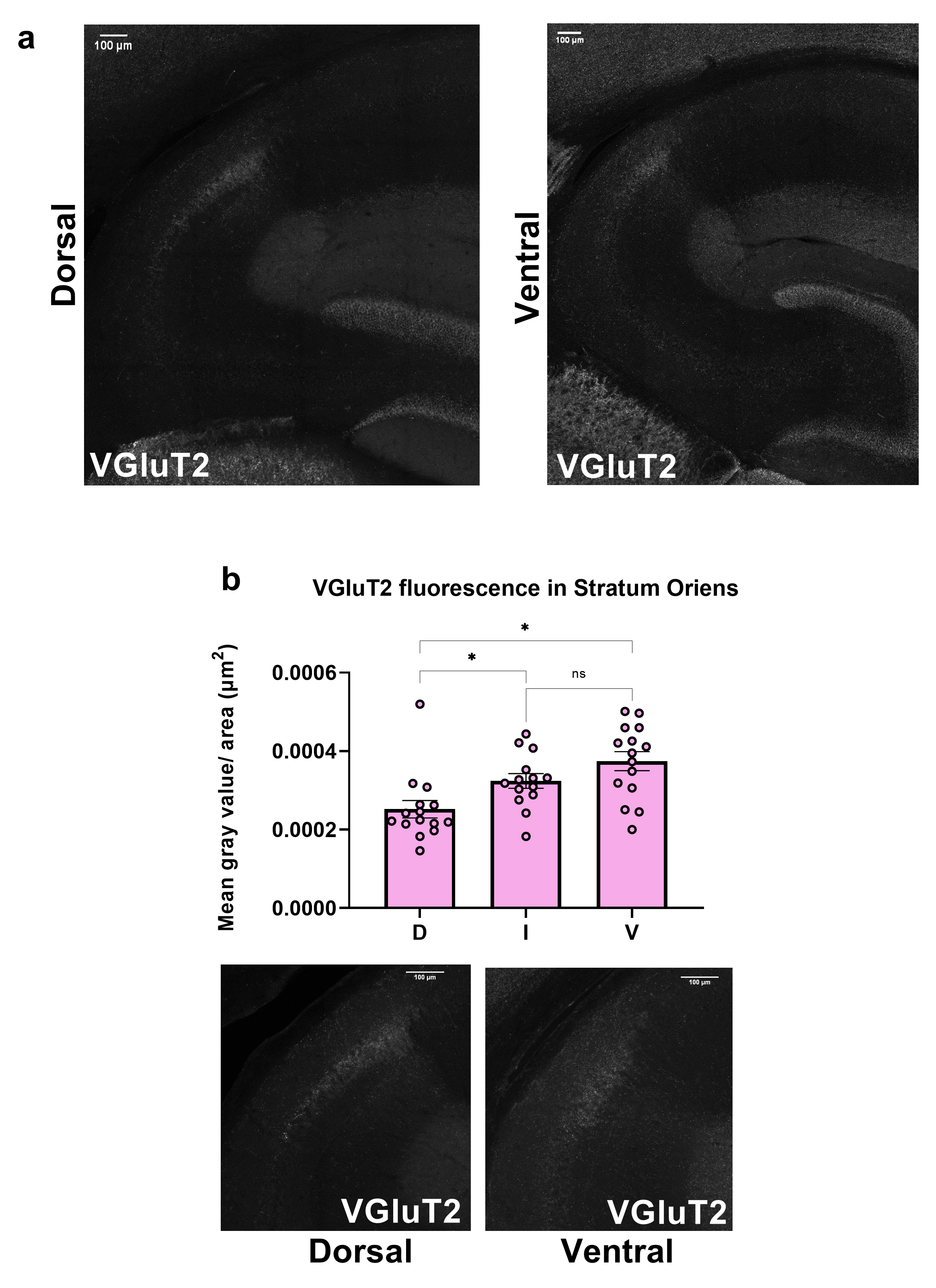

Supplement: Supplementary file 5 — FIGURE S5: Dorsoventral differences in the labeling pattern of VGluT2. (a) VGluT2 labeling in the stratum oriens extends diffusely into proximal CA3 in ventral (right), but not dorsal (left) depths. (b) The mean fluorescence intensity of VGluT2 labeling in stratum oriens is greater in ventral levels (bottom right; dorsal 0.00025 ± 0.00002 vs. intermediate 0.00032 ± 0.00002 p = .02, t = 2.46, f = 1.55; intermediate 0.00032 ± 0.00002 vs. ventral 0.00037 ± 0.00002 p = .11, t = 1.63, f = 1.83; dorsal 0.00025 ± 0.00002 vs. ventral 0.00037 ± 0.00002 p = .001, t = 3.72, f = 1.18, n = 44 sections total, 4–5 sections per dorsoventral depth from 5 animals). Scale bars = 100 μm. [file HIPO-33-133-s005.tif]
